# Low‐Temperature Nanosecond Laser Process of HZO‐IGZO FeFETs toward Monolithic 3D System on Chip Integration

**DOI:** 10.1002/advs.202401250

**Published:** 2024-05-13

**Authors:** Dongsu Kim, Heejae Jeong, Goeun Pyo, Su Jin Heo, Seunghun Baik, Seonhyoung Kim, Hong Soo Choi, Hyuk‐Jun Kwon, Jae Eun Jang

**Affiliations:** ^1^ Department of Electrical Engineering and Computer Science Daegu Gyeongbuk Institute of Science & Technology (DGIST) Daegu 42988 South Korea; ^2^ Department of Engineering Institute for Manufacturing University of Cambridge Cambridge CB3 0FS United Kingdom; ^3^ Department of Robotics and Mechatronics Engineering Daegu Gyeongbuk Institute of Science & Technology (DGIST) Daegu 42988 South Korea

**Keywords:** emerging memory devices, ferroelectric, IGZO‐HZO FeFET, laser annealing, low thermal budget, monolithic 3D integration

## Abstract

Ferroelectric field‐effect transistors (FeFETs) are increasingly important for in‐memory computing and monolithic 3D (M3D) integration in system‐on‐chip (SoC) applications. However, the high‐temperature processing required by most ferroelectric memories can lead to thermal damage to the underlying device layers, which poses significant physical limitations for 3D integration processes. To solve this problem, the study proposes using a nanosecond pulsed laser for selective annealing of hafnia‐based FeFETs, enabling precise control of heat penetration depth within thin films. Sufficient thermal energy is delivered to the IGZO oxide channel and HZO ferroelectric gate oxide without causing thermal damage to the bottom layer, which has a low transition temperature (<250 °C). Using optimized laser conditions, a fast response time (<1 µs) and excellent stability (cycle > 10^6^, retention > 10^6^ s) are achieved in the ferroelectric HZO film. The resulting FeFET exhibited a wide memory window (>1.7 V) with a high on/off ratio (>10^5^). In addition, moderate ferroelectric properties (2·P_r_ of 14.7 µC cm^−2^) and pattern recognition rate‐based linearity (potentiation: 1.13, depression: 1.6) are obtained. These results demonstrate compatibility in HZO FeFETs by specific laser annealing control and thin‐film layer design for various structures (3D integrated, flexible) with neuromorphic applications.

## Graphical abstract

This study employs a nanosecond pulsed laser to innovate low‐temperature processing for hafnia‐based ferroelectric field‐effect transistors (FeFETs), crucial for in‐memory computing and 3D system‐on‐chip integration. By selectively annealing with controlled heat penetration, it achieves enhanced device compatibility and memory performance without damaging the bottom layers, paving the way toward advanced monolithic 3D integration and flexible electronics.

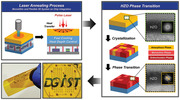

## 1 Introduction

Advances in electrical devices have produced a paradigm change in information storage and processing.^[^ ^1^ ^]^ Subsequently, these developments have increased the demand for memory structures capable of meeting the high technical requirements of various modern applications.^[^ ^2^ ^]^ In response, the research field has increasingly shifted toward the development of memory devices that employ Monolithic 3D (M3D) technology for System‐on‐Chip (SoC) processes, aiming to meet key specifications such as power efficiency, performance, reduced manufacturing cost, lightweight construction, and enhanced integration.^[^ ^3^ ^]^

Various advanced memory devices have subsequently emerged, including resistive random access memory (RRAM), phase change memory (PCM), charge trap transistors, and ferroelectric field‐effect transistors (FeFETs).^[^ ^4^, ^5^, ^6^, ^7^ ^]^ Among these advanced technologies, FeFETs in particular have received considerable recognition because of their non‐volatile memory characteristics, high‐speed operation, low power consumption, long lifespan, and durability.^[^ ^8^ ^]^ Ferroelectric materials for FeFETs can be realized by phase transformation through an annealing process and include PZT (lead zirconate titanate), SBT (strontium bismuth tantalum), PVDF (Polyvinylidene fluoride), and HfO_2_ (Hafnium oxide)‐based compounds.^[^ ^9^, ^10^, ^11^, ^12^ ^]^ Among these, HfO_2_‐based ferroelectrics are notable because of their ability to retain high ferroelectricity even when very thin. Accordingly, HfO_2_‐based ferroelectric materials have great potential for use in data‐intensive applications such as artificial intelligence, machine learning, edge computing, and in‐memory structures.^[^ ^13^, ^14^, ^15^ ^]^

Despite their high potential, current applications of HfO_2_‐based ferroelectrics in memory devices with M3D integration face limitations due to the high‐temperature annealing processes required, typically above 600 °C, which can cause thermal damage to underlying logic or input/output (I/O) circuits. To address this issue, various doping technologies, such as Zr, Al, Si, and Y, have been explored to enable phase transformation at lower temperatures via rapid thermal annealing (RTA).^[^ ^16^ ^]^ Notably, Zr doping is particularly promising due to its similarity to Hf and its ability to maintain ferroelectricity across a broad composition range and achieve higher remanent polarization than other dopants.^[^ ^17^ ^]^ Nevertheless, the annealing process in the 400 to 600 °C temperature range required for HZO films poses challenges for 3D integration.^[^ ^18^ ^]^ Additionally, the RTA process has difficulty in locally annealing, which can lead to performance degradation and thermal damage to the bottom layers of the device.^[^ ^19^ ^]^ To overcome these challenges, the development of specific partial heat treatment methods and lower thermal budget control processes is essential, ensuring the compatibility of diverse materials and structures for M3D integration.^[^ ^20^, ^21^, ^22^, ^23^ ^]^

In this study, we utilized a laser annealing process with a selective heat treatment application to anneal specific areas, addressing the challenges posed by high‐temperature processes. Our laser annealing system has a thin penetration depth (30 to 50 nm) because of the short wavelength (355 nm). Additionally, the fast‐cooling characteristics of the nanosecond‐pulsed (30 ns) laser system also help efficiently induce phase transformations, which are essential for facilitating the ferroelectricity of Hafnium Zirconium Oxide (HZO). The FeFET memory devices were fabricated in a sequentially integrated process system based on the phase formation of HfO_2_‐based ferroelectrics and activation of channel materials using low‐thermal budget annealing. Optimized FeFETs with extended properties for neuromorphic applications were studied. Additionally, we conducted experiments on a thermoplastic polyimide (PI) with a low transition temperature (<250 °C) to demonstrate the advanced local annealing process essential for M3D SoC integration. Mechanical, electrical, and chemical characteristics were analyzed by adjusting the layer thicknesses in the device to ensure stability under laser annealing conditions. The optimized properties of the Indium Gallium Zinc Oxide (IGZO)‐HZO Fe‐FET devices fabricated with low thermal budget laser annealing are expected to drive innovation in M3D applications for SoC integration technologies, and the results demonstrate the potential to expand into new architectural areas in the future.

## 2 Result and Discussion

### 2.1 Laser Set‐Up and Device Fabrication for monolithic 3D Integration Processes

**Figure 1:**
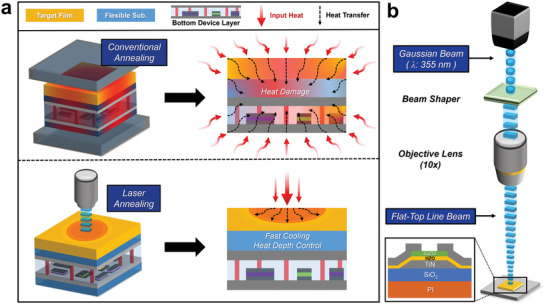
Annealing method for M3D integration processes a) Schematic illustration comparing the heat distribution in multi‐layer devices between laser annealing and conventional annealing. b) Schematic of the laser system with device structure.

**Figure** **1**a is a schematic illustration showing the difference in heat distribution characteristics of conventional and laser annealing processes. Conventional annealing methods (including furnace and RTA process) can cause thermal damage to other layers due to their random heat distribution and wide‐range wavelength energy distribution characteristics. On the other hand, the nanosecond pulse laser annealing method generates pulses of 30–40 ns, which raise the surface temperature quickly and then allow it to cool rapidly, enabling localized annealing (Figure S1a, Supporting Information).^[^ ^24^, ^25^, ^26^ ^]^ Additionally, such laser annealing systems use a single wavelength, allowing more precise optimization of the thermal budget based on material absorption properties.^[^ ^27^ ^]^ As a result, laser annealing can effectively manage the thermal budget, ensuring optimal temperature control in complex 3D and flexible devices incorporating multi‐material layers.

Figure 1b shows a schematic diagram of the laser device used in the experiment. The laser beam used in the experiment was a flat‐top line beam that converted the Gaussian beam through a beam shaper. The shape of the designed beam (150 µm × 10 µm) is shown in Figure S1b (Supporting Information). This design enhances the uniformity of heat distribution over the laser scanning area.^[^ ^28^, ^29^ ^]^ To demonstrate the advantages of nano‐pulse laser technology in preserving the integrity of the underlying layer, the device was fabricated with a polyimide substrate that has a low transition temperature (<250 °C). Figure S2 (Supporting Information) demonstrates the extent of surface degradation observed on the low‐transition temperature polyimide when subjected to RTA conditions at 250 °C for 3 min. The fabrication process of the device is depicted in Figure S3 (Supporting Information). As illustrated in Figure S3a (Supporting Information), the device comprises a multi‐layered thin‐film structure consisting of HZO/Titanium‐nitride (TiN)/Silicon‐dioxide (SiO_2_) as the bottom gate on a polyimide substrate. Prior to annealing for HZO crystallization, a 50 nm TiN assistant layer is deposited. Double‐sided forming with TiN/HZO/TiN induces stress between TiN and HZO during annealing, promoting a low thermal budget and resulting in high‐quality ferroelectric HZO films.^[^ ^30^, ^31^ ^]^ After the laser annealing process, the top electrode made of a 50 nm TiN film is removed using an H_2_O_2_ solution in a wet etching process. In the device structure, the SiO_2_ bottom layer functions as the Interlayer Dielectric (ILD) and Inter Metal Dielectric (IMD) layers in M3D with SoC architectures. This has the effect of minimizing leakage current and noise, dissipating thermal energy, and providing mechanical protection.^[^ ^32^, ^33^ ^]^ Atomic force microscope (AFM) confirmed that a 100 nm thick SiO_2_ layer provides effective thermal insulation and a high‐quality flat surface (R_a_ = 0.28 nm, scan rate = 0.3 Hz, 25 µm^2^), as shown in Figure S3b (Supporting Information). Additionally, the properties of this SiO_2_ layer, along with precise beam shaping, optimize the control system environment for nanopulse laser annealing in M3D integrated processing.

### 2.2 Effect of Laser Parameters on HZO Film Properties

**Figure 2:**
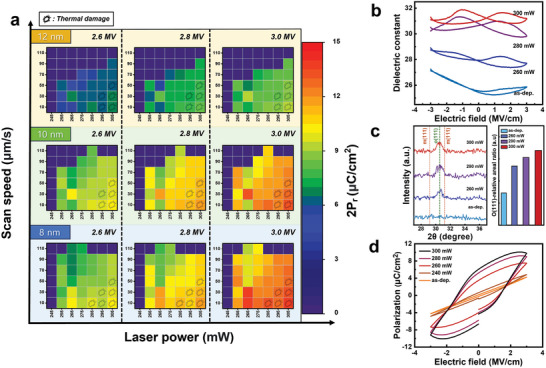
The impact of laser annealing parameters on HZO film properties a) The effects of HZO thickness and an applied E‐field on polarization data for TiN/HZO/TiN/SiO_2_/PI films annealed at various laser conditions (100 µm × 100 µm, 1 kHz) with thermal damage marks. b) Variation in dielectric constant with E‐field in TiN/HZO/TiN/SiO_2_/PI films annealed at different laser powers (100 µm × 100 µm, 10 kHz). c) Peak position and intensity distribution in glancing mode (with 3°) X‐ray diffraction of HZO/TiN/SiO_2_/PI annealed at varied laser powers with the relative areal ratio of orthorhombic phase (o‐phase/(o, t, m‐phase)). d) Polarization‐electric field curves in TIN/HZO/TIN/SiO_2_/PI with varying laser annealing powers (100 µm × 100 µm, 1 kHz, standard bipolar).

Using the previously described laser settings and multi‐layer structure, we first optimized the laser annealing conditions for HZO film. In **Figure** **2**, to optimize the laser annealing process conditions for ferroelectric HZO films, the polarization characteristics of the laser system on the TiN/HZO/TiN/SiO_2_/PI structures were investigated based on the scan speed and power parameters. To prevent thermal damage to the PI substrate, we aimed for the optimal balance between performance and stability instead of maximizing the 2P_r_ value. The marks in Figure 2a indicate thermal damage to the underlying layers. As shown in Figure S4a (Supporting Information), thermal damage in the PI layer was identified depending on the laser power and scan speed These observations demonstrate that ablation occurs as a result of excessive thermal expansion induced by laser absorption at the surface. The thermal damage depended on power rather than scan speed and appeared gradually at power levels exceeding 300 mW. These characteristics mean nanosecond pulse laser annealing systems require sophisticated laser optimization processes using an M3D integration process with diverse materials.

For effective operation, a FeFET device requires a wide memory window, high dielectric constant, and low current density.^[^ ^34^ ^]^ Among them, the memory window characteristics of the FeFET are significant operating parameters in memory applications.^[^ ^35^ ^]^ This means having HZO ferroelectric films with stable and high polarization properties is crucial. We used laser annealing on samples of various HZO thicknesses (8, 10, 12 nm) to optimize the device in Figure 2a. These results show that thinner HZO films tend to have higher polarization values. Typically, for HZO ferroelectric films deposited on TiN electrodes via atomic layer deposition (ALD), the polarization properties improve when the thickness is reduced to a specific value.^[^ ^36^ ^]^ Although this unique phenomenon has not been widely studied, the mechanism for improving TiN‐HZO film ferroelectric properties is believed to be tensile stress and switching‐leakage current phenomena.^[^ ^37^, ^38^ ^]^ This also suggests that higher laser powers tend to have relatively higher polarization. As the COMSOL simulation results in Figure S4b,c (Supporting Information) confirm, higher laser power provides the amount of thermal energy required for phase transformation in the TiN/HZO/TiN structure. The results of the simulations also revealed that precise control of the laser power would result in the temperature range (>400 °C) needed to induce HZO ferroelectricity. Furthermore, during the rapid heating and cooling process of laser annealing, tensile stress is reinforced due to the disparate thermal expansion coefficients of TiN and HZO, facilitating more efficient ferroelectric phase transformation.^[^ ^39^ ^]^ Figure S4c (Supporting Information) shows the thermal diffusion characteristics observed in TiN/HZO/TiN/SiO_2_/PI structures under different laser power conditions. This was achieved using a nanosecond pulse laser and effectively managing heat transfer through the TiN/SiO_2_ sublayer to the PI under‐layer, which significantly reduced thermal diffusion. This allows precise control of the required thermal budget during annealing. Based on the results, a 30 ns pulsed 355 nm short‐wavelength laser annealing system would potentially enable localized annealing.

Higher polarization properties could be obtained using thinner HZO films and higher laser power. However, the potential for crack formation due to the high thermal energy may also increase. This would reduce film stability because of excessive stress and switching leakage currents that exceed the mechanical limits of the thin film. Therefore, the measurement conditions in Figure 2b–d were optimized to a speed of 60 µm s^−1^ with 10 nm HZO film, resulting in a relatively crack‐free film for a more stable device and better system integration. In FeFET devices, dielectric leakage current significantly impacts information retention, stability, response speed, and power consumption.^[^ ^40^ ^]^ Additionally, if the breakdown point is too low, the function generator becomes susceptible to transient overshoots. This is a crucial consideration in memory system operation. Although the 8 nm HZO film showed relatively excellent polarization characteristics because of laser annealing, the thickness was optimized to 10 nm, considering both leakage current and device stability. Figure 2b presents the dielectric constant under different annealing conditions for the optimized (60 µm s^−1^, 10 nm HZO film) sample thickness. Non‐annealed samples do not have the butterfly curve characteristic of ferroelectric properties. However, as the laser power increases, the dielectric constant increases with a butterfly‐shaped curve that is more clearly visible. At higher power levels (300 mW), it shows a distinct butterfly curve with a high dielectric constant (ε_r_ ≅ 31.8). This effect is primarily due to the increased orthorhombic (o)/tetragonal (t) phase ratio in the HZO film.^[^ ^41^ ^]^ The higher the dielectric constant, the more advantageous it is for scaling, low‐voltage operation, and data preservation, enabling low‐power consumption devices to be implemented.^[^ ^42^ ^]^

To confirm the crystallinity of the HZO ferroelectric film in response to laser power, the peak position and intensity (θ−2θ) data of glancing mode X‐ray diffraction were analyzed, as shown in Figure 2c. In the non‐annealed films, the investigated samples did not show HZO ferroelectric properties at the crystalline peak corresponding to the o‐phase (30.5°).^[^ ^43^ ^]^ However, as the power was gradually increased from 250 to 300 mW, the emergence of a distinct crystalline structure was confirmed by X‐ray diffraction analysis (XRD) peak intensity as o, t, monoclinic (m) ‐phase. It can be seen that the relative areal ratio of the orthorhombic phase (o‐phase/(o, t, m‐phase)) according to each laser condition increases proportionally as power increases. Additionally, the width of the peaks tends to increase. This phenomenon is due to the formation of t/o‐phase and m‐phase peaks ≈28.5°, 30.5°, and 31.6° in the Glancing mode (with 3°) (GI)‐XRD spectrum.^[^ ^44^ ^]^ Moreover, a peak ≈27.5° was attributed to rutile TiO_2_ (110) because excessive thermal energy induces an active interfacial reaction between TiN and HZO.^[^ ^45^ ^]^ Figure 2d shows the polarization‐electric field relationship as the laser power on the sample was systematically increased under the conditions (60 µm s^−1^, 10 nm) optimized for the mechanical and electrical stability of HZO. The results confirmed that the highest polarization characteristics were achieved at high laser power (300 mW). However, at too high of a power, the laser‐annealing process cause unnecessary oxide formation, cracking, and peeling of certain film parts. Under the optimized conditions, 280 mW is a reasonable power, striking a balance between sacrificing some polarization characteristics and achieving high stability. Different electrode and sub‐layer choices can reduce these thermal budget issues. However, completely solving the thermal budget problem is complex. Additionally, TiN electrodes also contribute to the ferroelectricity of HZO films through interfacial reactions and lattice mismatch constraints.^[^ ^46^ ^]^ These results show that annealing the HZO film at a power threshold of ≈280 mW ensures optimal crystallinity and device stability in the TiN/HZO/TiN/SiO_2_/PI structure. These observations highlight the need to optimize the laser annealing process by considering the material properties and the integrity of the measurement procedure.

We comprehensively investigated the potential to tune the remanent polarization (P_r_) properties of the laser‐annealed HZO thin films through laser power changes, scan speed variations, and film thickness adjustments for advanced multi‐layer integration in logic and system applications. The excellent local annealing properties achieved by the laser system also suggest it is possible to achieve more precise control by optimizing the materials.

**Figure 3:**
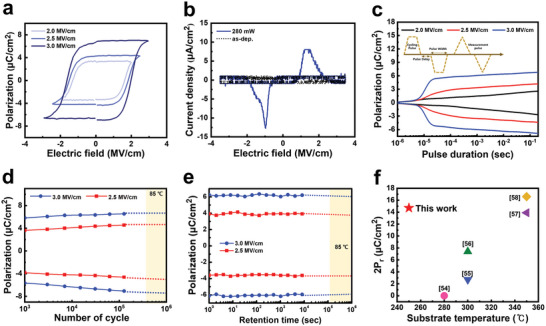
Characteristics of laser‐annealed TiN/HZO/TiN/SiO_2_/PI device a) Polarization characteristics of 280 mW laser‐annealed TiN/HZO/TiN/SiO_2_/PI sample with incremental electric field (100 µm × 100 µm, 1 msec, PUND measurements). b) Influence of 280 mW laser annealing on current density during electric field sweep (100 µm × 100 µm, 1 kHz, standard bipolar). c) Time‐dependent remnant polarization (P_r_) analysis under different pulse amplitudes (2.0, 2.5, 3.0 MV cm^−1^) in PUND measurements. d) Analyzing endurance characteristics through cycle‐remanent polarization relationship under varied pulse amplitudes (2.5, 3.0 MV cm^−1^) in PUND measurements. e) remanent polarization retention behavior under varied pulse amplitudes (2.5, 3.0 MV cm^−1^) in PUND measurements. f) The 2·P_r_ substrate temperature benchmark for HZO in other previous works.

**Figure** **3** shows the results of the detailed investigation of the polarization properties under HZO laser annealing conditions (280 mW, 60 µm s^−1^, 10 nm) to optimize memory application performance while maintaining operational stability. Figure 3a shows a pronounced polarization response that increases beyond the coercive field. Through Positive‐Up‐Negative‐Down (PUND) measurements, we observe that the remanent polarization values correspondingly increase as the electric field magnitude is raised. The interaction between polarization and electric field strength allows various memory states to be achieved, enhancing the efficiency of various memory applications.^[^ ^47^ ^]^ The hysteresis effect of the ferroelectric material is also shown in Figure 3b with the current density of the thin film. The observed high switching recent peaks indicate high polarization characteristics.^[^ ^48^ ^]^ To confirm the microscale uniformity of the optimized samples, polarization was measured using contact resonance piezoresponse force microscopy (CR‐PFM). A ±3 V programmed area utilizing the Piezoresponse Force Microscopy‐Electrostatic Force Microscopy (PPP‐EFM, k ≈2.8 N/m, resonance frequency: 75 kHz) mode was scanned using a −0.3 V tip bias. The measurement results as PFM phase, are shown in Figures S5 (Supporting Information). Based on a 20 µm bar‐line with 256 data points, we can observe stable and reproducible results even at nano level data point scale. In Figure 3c–e, polarization induction was observed through input pulses with various electric fields below the breakdown point to ensure stable measurements. To optimize the memory operation of the device, we conducted polarization measurements while varying the durations of input pulses, as illustrated in Figure 3c. These measurements were performed using the PUND configuration to examine polarization characteristics.^[^ ^49^ ^]^ The polarization characteristics were observed to vary with the applied electric field. When the duration value remained constant, higher electric field strengths led to more pronounced changes in polarization. Additionally, since the linear range of polarization duration varies for different electric fields, it can be seen that different voltages and durations must be used to achieve linear incremental polarization. These characteristics, in alignment with Equation (1), indicate there is a notable correlation between the voltage and the pulse duration needed to induce polarization in the ferroelectric material.

(1) $$P\left(t\right)=P_{s}\left\{1-2\exp\left[-\frac{t}{t_{s}}\exp{\left(-\frac{E_{d}}{V/d_{FE}}\right)}^{n}\right]\right\}$$

In Equation (1), the variables are defined as follows: P_r_ represents the intrinsic or spontaneous polarization, E_d_ designates the activation electric field, d_FE_ corresponds to the thickness of the ferroelectric film, “n” serves as a constant used to describe the nucleation mechanism, and t_S_ signifies the switching time. This equation demonstrates the potential to precisely control the polarization switching process utilizing voltage pulses with tunable amplitude. Moreover, ferroelectric films can potentially respond even to nanosecond pulses if the appropriate amplitude is chosen. However, with such short‐duration high‐voltage pulses, one must be careful about the overshoot phenomenon.^[^ ^50^, ^51^ ^]^

To ensure the stability of memory devices, Polarization‐Cycle measurements were performed under an applied electric field, as illustrated in Figure 3d. For the examination of characteristics in the 10 nm ferroelectric HZO thin film over multiple cycles, a slight increase in polarization was observed at subsequent initial times. This phenomenon, known as “ferroelectric wake‐up,” can complicate system design and pose challenges to stability and efficiency, especially under severe conditions.^[^ ^52^ ^]^ The wake‐up effect in the results tended to reach a saturation point after a certain number of cycles. In the process, performance was improved compared to the initial state, which is helpful for low‐power operation. In Figure 3e, retention measurements show that the ferroelectric materials retain memory data for long periods of time. Accelerated thermal aging tests for cycle and retention measurements were performed at 85 °C.^[^ ^53^ ^]^ These results show that the ferroelectric thin films optimized by laser annealing have potential for memory device applications. As shown in the benchmark figure in Figure 3f, a record 2·P_r_ of 14.7 µC cm^−2^ was achieved under the low thermal budget of 250 °C.^[^ ^54^, ^55^, ^56^, ^57^, ^58^ ^]^ Typically, substrate temperature parameters limit the selectivity and properties of the material. However, the performance parameters in this study confirm that laser annealing can result in relatively high performance despite offering a low thermal budget process. The results demonstrate that the annealing process using a nanosecond pulse laser system with a short wavelength (355 nm) has the potential to perform sequential processes without damage to the bottom device layer.

### 2.3 Hysterisis Effect of HZO‐IGZO FeFET with Memory Characteristics

**Figure 4:**
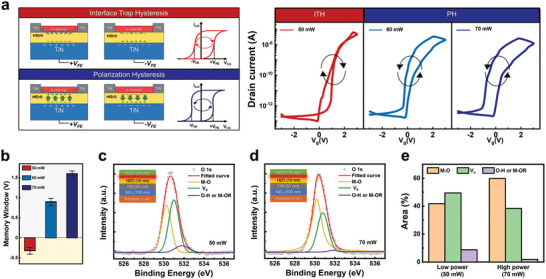
The hysteresis effect under various laser power conditions a) Working mechanism of IGZO‐HZO FeFET and transfer curve characteristics at various laser powers (W:L = 1:1, 50–70 mW, 60 µm s^−1^). b) Bar graph comparison of memory window sizes under various laser power conditions. c) XPS fitting analysis with deconvolution peaks of the IGZO/HZO interface in 50 mW laser‐annealed samples. d) XPS fitting analysis with deconvolution technique of the IGZO/HZO interface in 70 mW laser‐annealed samples. e) Bar graph comparison of M─O, V_o_, and O─H bond distributions from XPS fit data for each sample. f) Impact of laser annealing oxygen bonding on the IGZO films.

After successfully achieving the phase transformation of HZO through laser annealing, we optimized the laser conditions to activate the IGZO channel. **Figure** **4**a is a schematic diagram showing the hysteresis characteristics of the FeFET according to various mechanisms and the transfer curve results of the IGZO‐HZO FeFET annealed at different laser powers. In conventional n‐type FETs, the charge trap effect has a clockwise operating characteristic, as the red curve shows because of the positive V_th_ shift.^[^ ^59^, ^60^ ^]^ In contrast, the ferroelectric polarization effect has a counterclockwise characteristic, as shown by the blue curve, because of the effect of the remanent polarization field.^[^ ^60^, ^61^ ^]^ The transfer curve of the 50 mW annealed IGZO‐HZO FeFET is mainly influenced by the charge trapping effect rather than the polarization effect, while the 60 and 70 mW annealed FeFETs properties are affected by polarization rather than charge trapping. This suggests that the relative degrees of the charge trap effect and ferroelectric polarization effect can be adjusted depending on the intensity of the selected laser output. The memory window values for each power investigated (50, 60, and 70 mW) are represented by a bar graph in Figure 4b. For 50 mW, the clockwise hysteresis is −0.32 V (±3 V, V_g_), while for 60 and 70 mW, the values are +0.67 and +1.22 (±3 V, V_g_), respectively. The results show that the counterclockwise hysteresis window widens as the laser power increases. The presence of an optimal point for charge‐trap hysteresis in response to laser power can also be confirmed in the gate oxide TFTs with non‐ferroelectric HfO_2_ (10 nm), as shown in Figure S6 (Supporting Information). These results confirm enhanced on‐current and Vth stability through laser annealing of the IGZO channels within the IGZO‐HfO_2_ layer structure. This reveals the crucial role of laser power in optimizing the performance and interface of IGZO channels. The differences between the two devices result from the distinct physical and thermal properties of the HZO and HfO_2_ layers. The consistent behaviors observed in both results validate the effectiveness of the laser annealing process.

To enable a more comprehensive analysis, X‐ray photoelectron spectroscopy (XPS) was conducted following laser annealing of the IGZO film (Figure 4c,d). By spectral fitting the XPS data to determine the composition and chemical states of IGZO, the chemical bonding groups were classified as M─O (Metal‐Oxygen), V_o_ (Oxygen Vacancies), and O─H (Oxygen‐Hydrogen) bonds based on their respective oxygen binding energies at 530, 530.9, and 532.1 eV.^[^ ^62^, ^63^ ^]^ The ratios for each sample are compiled in the bar graph illustrated in Figure 4e to provide a quantitative analysis of each convoluted peak.

The bar graph clearly illustrates the transition from low‐power to high‐power laser annealing. The M─O bond increases from 41.8% to 59.8%, the V_o_ bond decreases from 49.4% to 38.3%, and the O─H bond decreases from 8.7% to 1.8%. Typically, when the M‐O bond ratio is low and the O─H ratio is high, it suggests there is inadequate bonding between the metal and oxygen, resulting in the presence of oxygen vacancies. Such cases lead to an increase in the overall leakage current.^[^ ^64^ ^]^ A high O─H ratio implies the interaction of hydrogen with oxygen, leading to an increased presence of oxygen traps because of hydrogen. The higher number of trap sites generated can significantly influence the device's electrical characteristics.^[^ ^65^ ^]^ An increased concentration of oxygen vacancies at the interface can lead to compromised electrical performance and stability because of alterations in the metal‐oxygen reactivity.^[^ ^66^ ^]^ According to the XPS results, high‐power laser annealing significantly contributes to reductions in O─H and V_o_ ratios, thereby mitigating trap formation. Increasing the laser power significantly impacts the reduction of O─H bonds and oxygen vacancies due to higher thermal energy.^[^ ^67^ ^]^

Although higher‐power laser annealing reduces IGZO trap sites, the optimal power level for IGZO annealing was determined to be 70 mW, representing a trade‐off between on/off performance and device stability. From the perspective of interface engineering, the laser annealing system enables precise modification of properties through localized annealing. This ability holds substantial promise for fabricating multi‐characteristics in memory, logic devices, and sensors.

**Figure 5:**
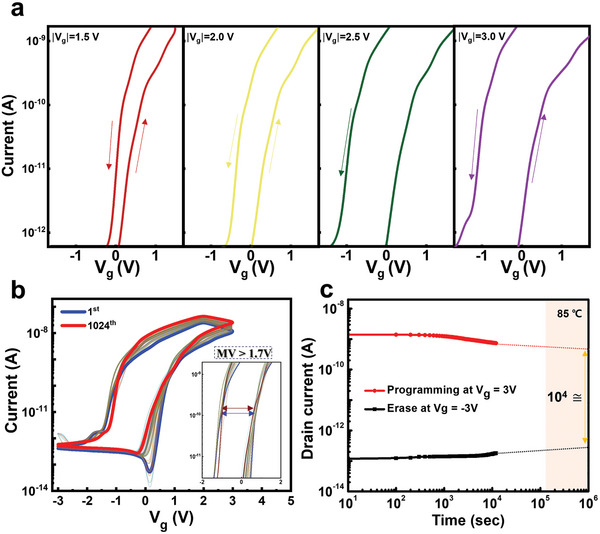
Performance characteristics of the optimized IGZO‐HZO Fe‐FET a) Transfer curves with incremental gate voltage sweep for optimized IGZO‐HZO Fe‐FET (V_DS_ = 1 V). b) Cycling test revealing transfer curves through gate voltage sweep (V_DS_ = 1 V). c) Retention analysis of IGZO‐HZO FeFET under accelerated measurements with sufficient pulse duration (V_DS_ = 0.7 V, 200 µs).

The properties of an IGZO‐HZO FeFET fabricated sequentially using a laser annealing system were investigated to confirm its potential as a memory application device. **Figure** **5**a details the incremental memory window observed during a gradual gate voltage sweep. Activating memory functions at specific voltage thresholds can significantly increase pattern recognition efficiency in neuromorphic systems.^[^ ^68^ ^]^ Unlike other two‐terminal synapse structures, the transistor‐type neuromorphic device has a considerably lower off current of pico‐ampere level when the off state, which helps reduce power consumption during inactive states. The memory window can expand to a width of up to 1.7 V, as demonstrated by the ±3 V gate voltage sweep. The degree of expansion relies on the amplitude of the gate voltage sweep, with the increase in memory window following a non‐linear relationship, as indicated by the polarization equation.

Figure 5b shows the electrical characteristics of on/off operation during the 1024^th^ gate voltage sweep. This gate sweep process can be observed as a change that is induced by applying electrical stress stronger than the single‐pulse gate input. The durability of the device was confirmed through a cycling test, and the inset figure shows an increase in on‐conductance compared to the initial state after repeated operation, which is due to the wake‐up effect of the HZO ferroelectric film. This effect is noticeable upon initial setup but reaches a saturation point after enough cycles and does not cause problems during neuromorphic operation. A relatively moderate memory window (1.7 V) was achieved at a low thermal budget of 250 °C.^[^ ^69^, ^70^, ^71^, ^72^ ^]^ The input level maintenance characteristics of the device were confirmed by performing an acceleration test in an 85 °C environment after measurements at 10 s intervals, as shown in Figure 5c. Although the on/off window decreased over time, the results showed a stable window of 10^4^ or more was secured even after sufficient acceleration measurement (≈10 years, >10^6^ s). The robustness of the HZO ferroelectric‐based Fe‐FET was verified through cycling and retention measurements. These characteristics are critical parameters to ensure operating reliability in memory applications.^[^ ^73^ ^]^

**Figure 6:**
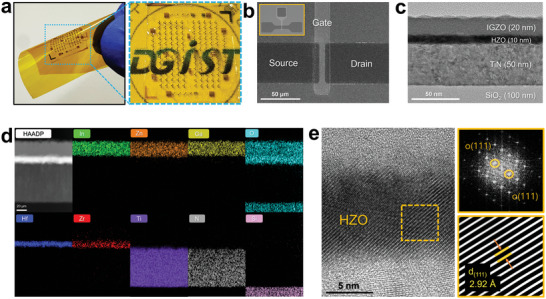
The configuration of the IGZO channel bottom gate HZO‐based FeFET device fabricated using laser annealing. a) Optical image of the sample HZO‐IGZO FeFET with polyimide used for M3D integration process verification. b) Top view SEM image indicating the active area, gate, drain, source, and body pads. c) HR‐TEM image of the channel stack, showing the thickness of each layer. d) EDS element mapping of the channel stack (PI/SiO_2_/TiN/HZO/IGZO). e) The HR‐TEM image of the laser crystallized TiN/HZO/IGZO stack and insert images are the FFT and inverse FFT patterns, corresponding to the orange frame.

**Figure** **6** shows the top view field emission scanning electron microscope (FE‐SEM) and cross‐sectional high‐resolution transmission electron microscopy (HR‐TEM) images of the fabricated IGZO channel bottom gate HZO‐based FeFET device prepared with laser annealing. The laser annealing conditions used were those previously confirmed as optimal, with laser powers of 280 and 70 mW used for the HZO and IGZO thin films, respectively, and a scan speed of 60 µm s^−1^. Figure 6a shows an optical picture of multi‐cell IGZO‐FET integrated with polyimide to verify the M3D integration process. A top‐view of SEM image in Figure 6b shows the ≈10% overlap exists between the source, drain, and gate regions. The cross‐sectional TEM images in Figure 6c show the uniform deposition of each thin film stack in the fabricated device. In Figure 6d, the Energy‐Dispersive X‐ray Spectroscopy (EDS) analysis indicates the presence of various elements including In, Zn, Ga, O, Hf, Zr, Ti, N, and Si. Significantly, these elements are not diffused in the HZO‐based FeFET device. Additionally, the EDS analysis confirms the stable super‐cycle deposition of Hf and Zr. This super‐cycle reduces the thermal budget required for hafnium‐based ferroelectric grains and has high stability.^[^ ^74^ ^]^ Figure 6e shows an HR‐TEM image of the HZO region from Figure 6c. The HR‐TEM image before laser annealing is presented in Figure S7 (Supporting Information) with a unit cell of HZO. In contrast, the laser annealing process resulted in a distinct transformation of the amorphous HZO thin film into a crystalline state throughout its entire thickness (10 nm). A fast Fourier transformation (FFT) diffractogram was obtained from the selected HZO region delineated by the orange frame. The observed lattice fringes, obtained through the inverse FFT analysis, were precisely indexed to the orthorhombic phase, specifically the (111) plane, distinguished by an interplanar spacing (d‐spacing) of 2.92 Å.^[^ ^75^ ^]^ These findings align with the XRD analysis presented in Figure 2, where the predominant o(111) peak observed following laser annealing substantiates the development of a ferroelectric phase. These results confirmed the stability of the film state of the IGZO‐HZO FeFET manufactured through sequential processes. The results also demonstrate the potential application of various materials to 3D‐stack and flexible structures without thermal damage to the bottom layer.

### 2.4 Enhancing Neuromorphic Functionality in IGZO‐HZO FeFETs

**Figure 7:**
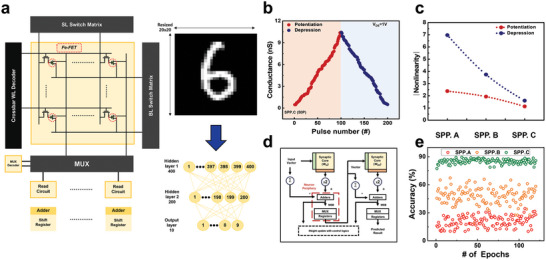
Exploring Various Aspects of Neural Network and IGZO‐HZO FeFET Behavior a) Artificial neural network structure for pattern recognition and comprehensive network setup. b) IGZO‐HZO FeFET conductance modulation using SPP.C pulse scheme for depression and potentiation. c) Nonlinearity comparison in IGZO‐HZO FeFET under different SPP pulse increment schemes. d) Circuit block diagram for hardware implementation in NeuroSim. e) Comparison of accuracy based on epochs using diverse programming pulses on measured device characteristics.

FeFET‐based artificial synapses were employed in the neuromorphic system introduced in **Figure** **7**a. This system is designed with an analog crossbar architecture and incorporates switch transistors that can access and modulate the weights of individual devices. It features multiple switch terminals for summary and weight updates to facilitate actual operation. In addition, unlike a two‐terminal structure, the synapse of the transistor structure can be expanded to a high level of neuromorphic system integration without a switching element. Therefore, it is important to explore approaches to neuromorphic systems that build on a thorough understanding of device properties. The optimized device characteristics were combined in the ANN+NeuroSim.V3 environment, a reliable benchmark simulator.^[^ ^76^ ^]^ In this system, the artificial neural network comprises 400 neurons in the input layer, 100 in the hidden layer, and 10 in the output layer. Adjustments to the network components and layers can impact performance when matching specific neuromorphic computing algorithms. The network design in this research followed the fundamental structure of backpropagation.

An investigation of the changes in conductance characteristics was conducted to evaluate the proven devices application as memory devices for neuromorphic recognition operations. The conductance of the FeFET can be changed based on Equation (1). Here, the pulse applied to the ferroelectric is defined by voltage and duration. Various types of pulse schematics for gate input optimization are introduced in Figure S8a (Supporting Information). All of the types initial and max voltages were set considering the device coercive voltage (1.5 V) and breakdown voltage (3.7 V).

The Identical Pulse Programming (IPP) type uses consistent voltage pulses, the Stepped Pulse Programming (SPP) type employs incremental voltage pulses, and the SPP.C type represents the pulse scheme optimized for this device. In this study, the voltage and duration of the pulse were strictly optimized to prevent excessive transient overshoot when using a function generator. Figure S8b (Supporting Information) explores the variation in conductance when using a consistent voltage pulse (200 µs). It was observed that the range of conductance change is rather limited when specific voltage levels are used. Higher voltage levels result in a broader range, while lower voltage levels lead to a significantly narrower range. However, high‐voltage pulses make accessing the lower‐level conductance range challenging, hindering a full‐range swing. This limitation can reduce the reliability of neuromorphic systems. Conversely, SPP. A and B (Figure S8c, Supporting Information) involve incremental voltage pulses (200 µs) with increment values of 0.08 V and 0.16 V. Both types allow full‐range conductance level changes. At large increments (0.16 V), the conductance level increases rapidly in the potentiation region. This phenomenon has a negative effect on linearity. Using a pulse type with a smaller increment results in more precise conductance changes. This increase in linearity significantly impacts the level of reliability in neuromorphic systems. Systems with higher linearity can achieve highly reliable recognition rates without additional power consumption.

In Figure S9 (Supporting Information), conductance changes were observed using continuous time control. Linearity was observed in the potentiation region with increasing duration. Pulse duration controlled by ferroelectric polarization mechanisms also showed the potential for high linearity. However, a duration control that is too low compared to system specifications can cause excessive overshoots. Therefore, we optimized the incremental voltage while focusing on device stability considerations in Figure 7b. The degree of linearity corresponding to the number of pulses, divided by increments, is depicted in Figure 7c. Specifically, it includes 25 pulses (SPP.A, 0.16 increment), 50 pulses (SPP.B, 0.08 increment), and 100 pulses (SPP.C, 0.04 increment). The linearity for each potentiation and depression, calculated through Figure S10 (Supporting Information), is reported as follows: (2.38, 6.97), (1.93, 3.73), and (1.13, 1.6), with values closer to 0 indicating ideal linearity.^[^ ^77^, ^78^ ^]^ Combining an optimized amplitude with duration can achieve a higher number of linear conductance level states, provided that systematic overshoot is prevented. The FeFET distinctive conductance change characteristics underscore its promising potential for application in neuromorphic models and computing systems.

In the simulation tasks, participants had to recognize established MNIST (Modified National Institute of Standards and Technology database) numerical data. The hardware implementation of a two‐layer MLP (Multi‐Layer Perceptron) neural network is depicted in Figure 7d, where the simulator focused solely on the main sub‐circuit modules surrounding the neurons during the weighted sum operation stage. Figure 7e shows the simulation outcomes, where the details of the artificial neural network were changed based on previous device measurements. The recognition confidence is depicted in terms of accuracy results across learning iterations (epochs). The results illustrate that SPP. C, characterized by relatively high linearity, exhibited significantly better accuracy (>88.72%) than the other two cases. In other words, the IGZO‐HZO FeFET manufactured through laser annealing shows a high recognition rate in neuromorphic operation based on optimized linearity through gate pulse scheme engineering.

Additionally, the PI substrate used to check the level of damage to the bottom layer exhibited potential value as a flexible device in Figure S11 (Supporting Information). Demonstrating the flexibility of the film, an inset that shows the structure is included in Figure S11a (Supporting Information). The high flexibility of the device in this study is enabled by the neutral‐plane theory presented in Figure S11b (Supporting Information). When an external force is applied to a film, it results in compressive stress on one side and tensile stress on the other. However, in the neutral plane within the material, the effects of these two stresses cancel each other out, resulting in zero net‐stress. In mechanical design, the location of the neutral plane is a crucial consideration, particularly when reducing the thickness of the PI coating on both sides for improved integration. The utilization of thin film device components (IGZO 20 nm, HZO 10 nm) offers distinct advantages, particularly their compatibility with small neutral layer regions. These thin components are useful as they can effectively operate within constrained neutral layer regions. Optimizing the neutral zone between the device and the substrate should improve device stability in specific applications where space constraints are considered. Spin‐coated PI films allow for precise thickness control to <1 µm. The COMSOL simulation results for the designed PI/FeFET/PI structure confirm that the device is in a safe blue zone (strain = 0).

Samples were configured into cylinders with varying radii to test the device's flexibility. The on/off operation at a gate voltage (V_g_) of 0.1 V is detailed in Figure S11c (Supporting Information). The results showed that the on/off ratio of FeFET was maintained despite various bending radii. These characteristics mean a neutral‐plane environment has been created, resulting in a device environment resistant to bending stress. Figure S11d (Supporting Information) shows the impact of bending time on the characteristics of on/off behavior (at V_g_ = 0.1 V) using the smallest radius cylinder (7.5 mm) employed during the measurement. As depicted in the inset image, the flexible device exhibited consistent behavior and maintained its characteristics without any changes over an extended duration while in a bent state. This observation underscores the effective performance of the neutral layer region as a reliable buffer to ensure device stability.

The results suggest that nanosecond laser annealing effectively solves the thermal budget problem and that this device has great potential as a flexible memory device. This offers an innovative solution for advanced M3D integration structures and flexible designs across diverse applications.

## 3 Conclusion

This study achieved a low process temperature using 355 nm nanosecond pulsed laser annealing FeFETs in a sequentially integrated process system to increase the compatibility of the hafnium‐based ferroelectric. By optimizing each device layer and utilizing local annealing without interference, we successfully achieved compatibility despite the large thermal mismatch between the HZO (>550 °C) and PI (<250 °C) materials. In addition, we demonstrated that activation in the oxide channel is achievable, and various transfer curves can be obtained only through laser annealing, without requiring any other post‐processing. This allows customization of properties depending on the laser setting. In addition, various thin film characteristics were achieved by precisely controlling laser conditions such as scan speed and power. These optimization methods suggest that it is possible to ensure compatibility among diverse structures and low transition temperature materials using laser annealing, in the selected area of next‐generation M3D integration. In conclusion, the proposed laser process applies to various 3D structures and flexible materials (polyester terephthalate, polyimide, polyester, and polyurethane) that require local annealing with a low thermal budget.

## 4 Experimental Section

### Laser System Setup

The 30 ns pulsed laser (wavelength: 355 nm, AVIA 355‐X, Coherent) was shaped as a line profile to deliver uniform energy to the substrate. The laser motion was controlled in the X‐Y‐Z directions by an Aerotech stage and actuators. To maintain focus, the height of the Z stage was adjusted using the stage program to fix the distance between the objective and the sample.

### Device Fabrication

The PI films were prepared by thickness and cleaned in acetone and Isopropyl alcohol (IPA) each for 20 min under ultrasonication. Afterward, a thin SiO_2_ film was deposited on the PI as an interfacial layer using an Radio Frequency (RF) sputter system. The TiN used as the electrodes and capping of the FeFET were laser deposited using a Direct Current (DC) reactive sputter with Argon (Ar) gas and Nitrogen. (N_2_) at a 6:4 ratio with 5 mTorr partial pressure and 100 mA power. Metal patterning was performed through a lift‐off process using acetone and IPA for 10 min each. The photoresist was removed with acetone/IPA/Deionised (DI) water. Afterward, the HZO film was deposited at 200 °C through plasma‐enhanced atomic layer deposition (PEALD, Lucida M200‐PL). TEMAH and CpZr were used as precursors based on the H_2_O reaction mechanism. The HZO super‐cycle was optimized for HfO_2_ and ZrO_2_ at 0.6 Å:0.4 Å. TiN used for capping was subjected to an etching process using hydrogen peroxide. The IGZO (In:Ga:Zn = 1:1:1) film used as the channel material was deposited at 20 nm through an RF sputter system at 100 W, 5 mTorr, Ar:O_2_ = 50:5 sccm. The gate contact pattern was fabricated by photolithography with an etching process. The pattern was spin‐coated with AZ nLoF negative photoresist at 3500 rpm and soft‐baked at 110 °C for 1 min. Development was performed with UV (365 nm) and an exposure energy of 70 mJ cm^−2^, and post‐exposure baking was performed at 110 °C for 1 min. The photoresist was developed using AZ‐300 developer for 1.4 min, and the samples were rinsed in DI water. Dry etching was carried out (P‐5000, 600 W, 11 s, CF_4_:SF_6_ = 50:50 sccm).

### Characterization (SEM, TEM, AFM, UV–vis–NIR, XRD, XPS)

To view the structure of the sample images were acquired through FE‐SEM (Hitachi/SU8230). To consider the film quality, the grid was measured with HR‐TEM (Thermo Scientific/Themis Z). To view the surface condition according to SiO_2_ thickness, measurements were made using an AFM (Park‐systems/XE‐150). To extract COMSOL simulation parameters, optical spectra, transmittance, and reflectance were measured from 200 to 800 nm using a UV–vis–NIR optical spectrometer (Cary 5000 UV–vis–NIR, Agilent Technologies, USA). An XRD analyzer confirmed the HZO phase (Panalytical/Empyrean). An XPS (Thermo Scientific/ESCALAB 250Xi) was used to observe the interface between IGZO and HZO.

### Electrical Measurements

A semiconductor parameter analyzer (Keithley, 4200‐SCS) equipped with a hot chuck stage system was used to determine the electrical properties of the FeFET. To extract neuromorphic characteristics, the measurement environment was configured with an arbitrary waveform generator (4225‐RPM, Keysight 33250A, B2902 Function) in Figure S12 (Supporting Information). The waveform was created using internal software (Easy EXPERT) and Labview programming. A ferroelectric tester (radiant, RT66C, Precision Premier series) was used to check the ferroelectric properties.

### Simulation

In Figures S13 and S14 (Supporting Information), simulations were performed using COMSOL Multiphysics software to analyze stress and laser annealing temperature distributions, which were some of the causes of mechanical failure. NeuroSim: A Circuit‐Level Macro Model was used to confirm pattern recognition accuracy, which varies depending on the performance of the synapse device. Each of the three layers (input, hidden, and output layer) consisted of 400, 100, 10 neurons. The performance was verified by learning a modified National Institute of Standards and Technology database (MNIST&eMNIST), a hand‐written digit dataset.

## Conflict of Interest

The authors declare no conflict of interest.

## Supporting information

Supporting Information

## Data Availability

The data that support the findings of this study are available from the corresponding author upon reasonable request.
