# Supplementary material for: Low‐Temperature Nanosecond Laser Process of HZO‐IGZO FeFETs toward Monolithic 3D System on Chip Integration
Source: Adv Sci (Weinh). 2024 May 13;11(28):2401250. doi: 10.1002/advs.202401250 (PMC11267387; doi:10.1002/advs.202401250)
Supplement: Supplementary file 1 — Supporting Information [file ADVS-11-2401250-s001.docx]

Supporting Information

**Low-Temperature Nanosecond Laser Process of HZO-IGZO FeFETs towards 3D Sequential Integration**

***Dongsu Kim^1^, Heejae Jeong^1^****, Goeun Pyo^1^, Su Jin Heo^1,2^, Seunghun Baik^1^, Seonhyoung Kim^3^, Hong Soo Choi^3^,* ***Hyuk-Jun Kwon******^1^*****,* ***Jae Eun Jang^1^****

^1^Department of Electrical Engineering and Computer Science, Daegu Gyeongbuk Institute of Science & Technology (DGIST)

^2^Department of Engineering, Institute for Manufacturing, University of Cambridge, Cambridge CB3 0FS, United Kingdom

^3^Department of Robotics and Mechatronics Engineering, Daegu Gyeongbuk Institute of Science & Technology (DGIST)

E-mail: jang1@dgist.ac.kr, hj.kwon@dgist.ac.kr

**Figure S1. Characteristics of nanosecond pulse laser annealing**

**Figure S2. Setting the bottom layer with a specific transition temperature**

**Figure S3. Fabrication flow of FeFET device**

**Figure S4. Observe the state of the sample according to the parameters of the laser system**

**Figure S5.** **Piezo-response force microscopy (PFM) with optimized HZO films**

**Figure S6. IGZO channel was optimized using IGZO-HfO_2_ stacking structure**

**Figure S7. Structural properties of laser annealing HZO thin films**

**Figure S8. Enhancing Neuromorphic Functionality in IGZO-HZO FeFETs**

**Figure S9. Type 4. Identical Pulse & Stepped duration Programming (IPP)**

**Figure S10. Linearity calculations for NeuroSIM tools**

**Figure S11. Structural and electrical properties of flexible memory**

**Figure S12. Measurement environment settings and programs**

**Figure S13. Analysis of optical properties of films to extract parameters to be used in simulations**

**Figure S14. Parameters for the materials in the functional layer used in simulation**

**Characteristics of nanosecond pulse laser annealing**


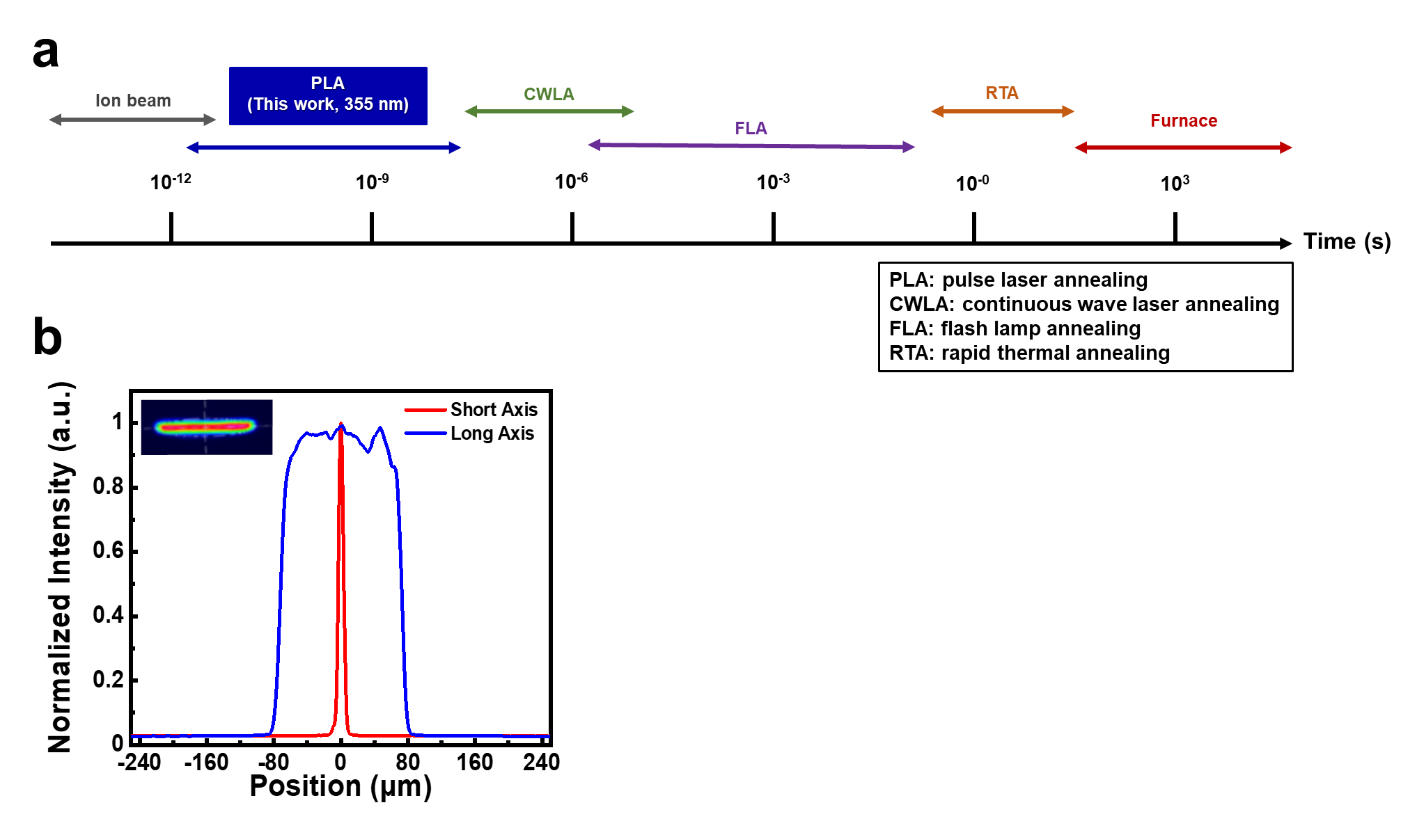


**Figure S1.** a) Comparison of time spectra for different annealing techniques (Ion Beam Annealing, Pulse Laser Annealing (PLA), Continuous Wave Laser Annealing (CWLA), Flash Lamp Annealing (FLA), Rapid Thermal Annealing (RTA), Furnace Annealing). b) Intensity graph of the flat-top line beam.

**Figure S1**a shows that selecting an annealing method with a shorter exposure time is more advantageous for local annealing. Additionally, commercial RTA equipment uses tungsten-halogen lamps as heat sources.[1] These sources have power intensity for all wavelengths. Because each material absorbs different wavelengths, the conventional annealing method (RTA, Furnace) has the disadvantage of making it difficult to control heat balance accurately. The flat-top line beam formed through a beam shaper and objective lens provides more uniform heat than a conventional Gaussian beam.

**Setting the bottom layer with a specific transition temperature**


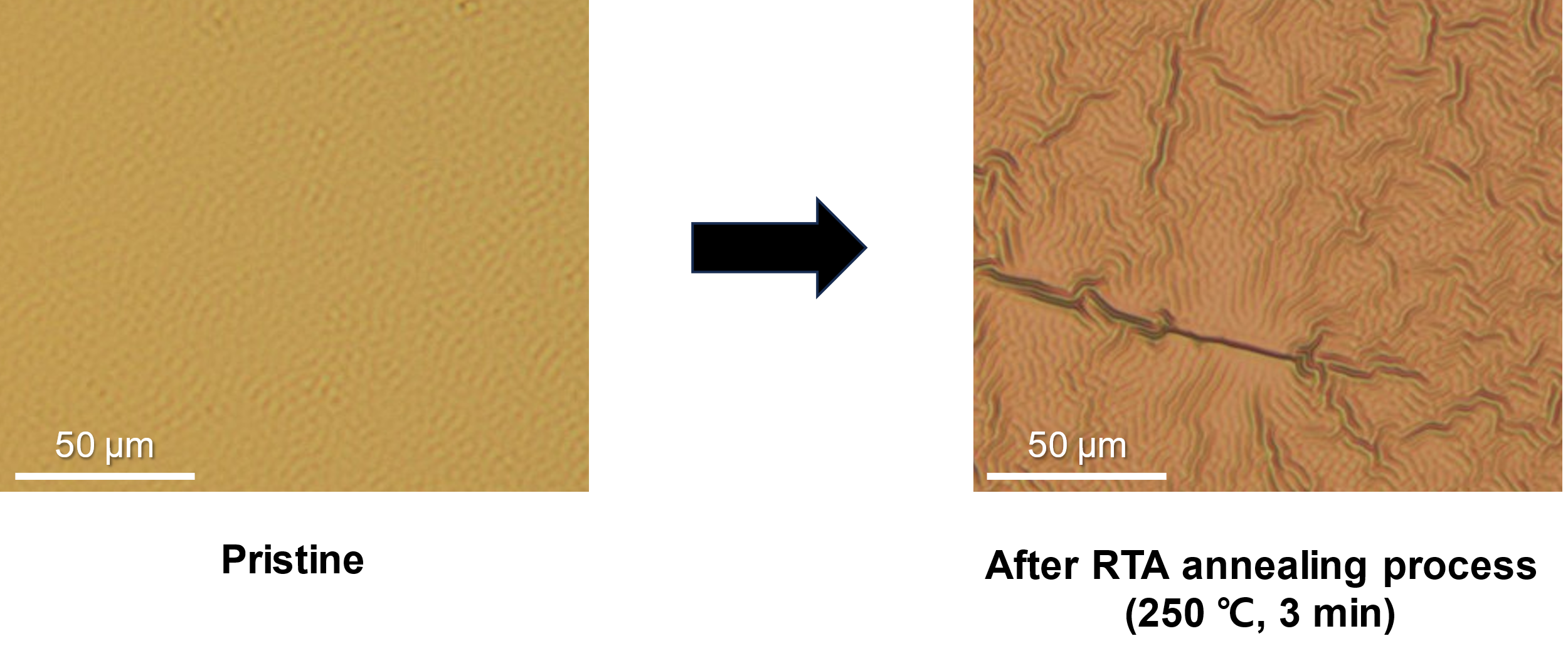


**Figure S2.** Optical images of the pristine to RTA annealing process of the bottom layer (Polyimide/SiO_2_/Si)

The device was constructed using Thermoplastic Polyimide (TPI-200), which has a relatively low transition temperature, in the bottom device area to verify strictly controlled heat distribution in the 3D integrated structure. To keep the TPI-200 film safe during the process, the HZO film used on top was deposited at 200 ℃. Additionally, the polyimide used has a relatively high light absorption, so it has the advantage of being able to observe the effect on laser annealing strictly. [2]

**Fabrication flow of Fe-FET device**

**
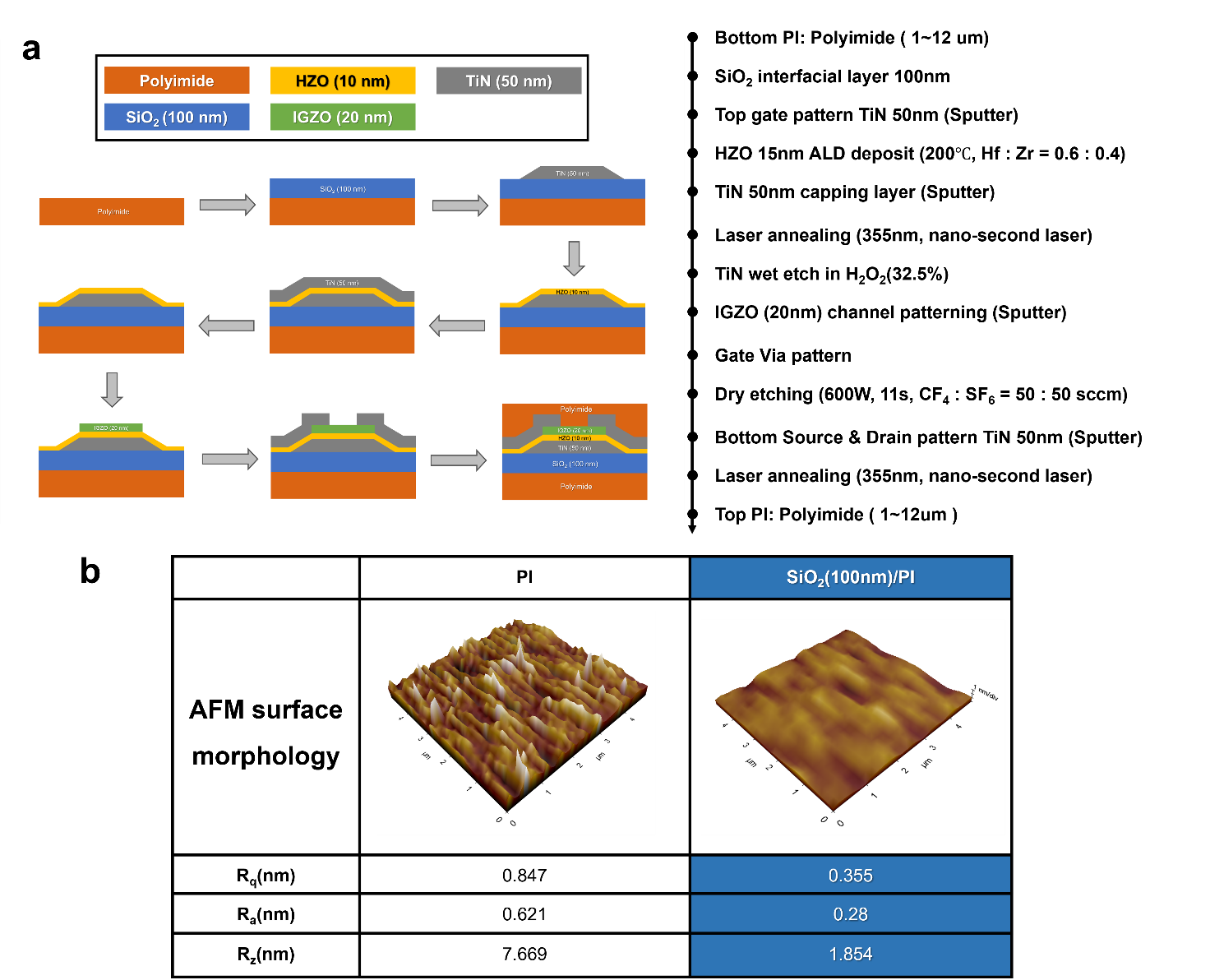
**

**Figure S3.** a) Structural overview of a FeFET with fabrication process flow. b) Comparison of surface roughness values (R_q_, R_a_, R_z_) obtained from Atomic Force Microscopy (AFM) measurements for different samples.

In the annealing process, using a nano pulse laser allows for localized heating. The laser affects a thin layer, about 30 to 50 nm deep.[3] The spin coating technique lets us apply a precise thickness of material on the carrier substrate, making the process consistent. We also find that different thicknesses of commercial polyimide (like TPI-200 from JCR) can be processed reliably. Initially, depositing silicon dioxide (SiO_2_) using RF-sputtering might damage the polyimide due to the plasma power. However, once we have a layer thicker than 10 nm, the process becomes stable.

Changing the ratio of components in the HZO creates ferroelectric films with different characteristics. In this study, using a super cycle with a 0.6:0.4 ratio helped reduce leakage currents. The top electrode stack also promotes ferroelectric properties in HZO at lower temperatures due to its capping effect. After laser treatment, we used hydrogen peroxide for etching in **Figure S3**a. We could open the areas for the source and drain electrodes using either buffer oxide etching or dry etching. The top layer of polyimide is chosen to match the physical properties of the bottom layer, creating a balanced structure. If different polyimides are used, it’s crucial to adjust their thickness to get the right physical properties.

Silicon dioxide is a key insulator in electronics, blocking unwanted currents between components. It also improves surface bonding and acts as a protective barrier against chemical reactions and mixing of materials. However, we need to carefully control its thickness to maintain device efficiency and reliability.[4] But as seen in **Figure S3**b, surfaces aren't smooth at thicknesses under 100 nm, and laser light scattering can affect this, so smoothing these surfaces is essential.[5]

**Observe the state of the sample according to the parameters of the laser system**

**
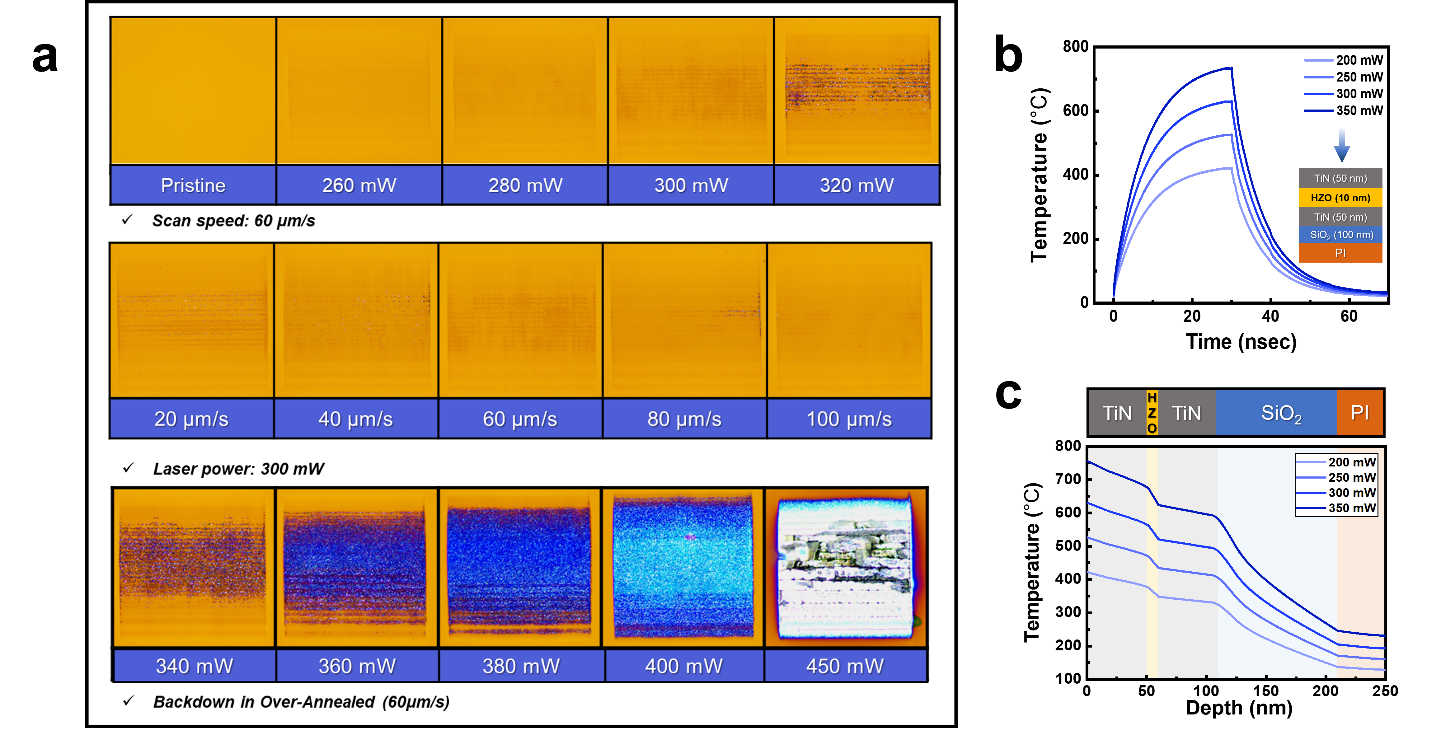
**

**Figure S4. Optical examination in various annealed conditions of TiN/HZO/TiN/SiO_2_/PI structure** a) Scan speed and laser power dependency. (b) Simulation results of surface (TiN/HZO/TiN) temperature variation with 355 nm pulse laser. (c) Thermal distribution through TiN/HZO/TiN/SiO_2_/PI structure.

**Figure S4**a illustrates the effects of adjusting the scan speed of the laser system. At excessively high speeds, maintaining uniformity at the edges of the irradiated area becomes challenging. Therefore, a careful balance between speed and uniformity is necessary in laser annealing systems. The figure also documents the occurrence of cracks and burning in the film due to excessive power. These observations indicate that damage to the underlying layers is predominantly influenced by the laser power rather than the scan speed. **Figures S4**b and **S4**c validate the annealing properties of TiN/HZO/TiN/SiO_2_/polyimide (PI) structures through COMSOL simulations under varying laser power conditions. In **Figure S4**b, surface laser annealing exhibits diverse temperature ranges, which is crucial for inducing HZO ferroelectricity (> 450 °C), with a rapid 30 ns heating and cooling cycle. **Figure S4**c illustrates thermal diffusion characteristics, shows the effectiveness of nano-pulse laser annealing in minimizing heat transfer to PI through the TiN/SiO_2_ sublayer. These findings emphasize the potential of 355 nm short-wavelength laser annealing systems for precise heat budget control and localized heating during annealing.

**Piezo-response force microscopy (PFM) with optimized HZO films**


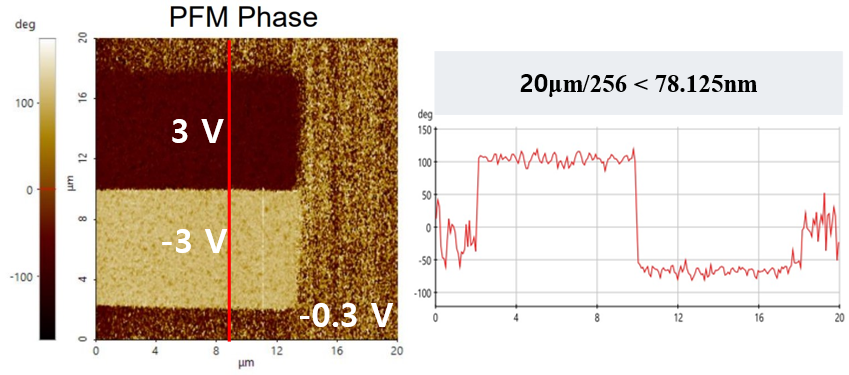


**Figure S5.** The backward polarized Piezo-response force microscopy (PFM) with optimized HZO films (HZO laser annealing conditions (280 mW, 60 μm/s, 10 nm)) amplitude difference images with a squared 20 × 20 μm^2^ area.

The HZO/TiN samples, optimized under laser annealing conditions (280 mW, 60 μm/s, 10 nm), were characterized using Contact Resonance Piezoresponse Force Microscopy (CR-PFM) in the Piezoresponse Force Microscopy-Electrostatic Force Microscopy (PPP-EFM, k ~ 2.8 N/m, resonance frequency: 75kHz) mode to define and program regions. The programmed areas, set at a voltage of 3V, were scanned using a tip bias of -0.3V. The resulting phase and amplitude of the regions, programmed at contrasting voltages, can be examined in **Figure S5**. This demonstrates not only the capacity for defining ferroelectric domains at the nanoscale (< 80 nm) but also provides evidence of the stability and repeatability of the ferroelectric response across the device.

**IGZO channel was optimized using IGZO-HfO_2_ stacking structure**

**
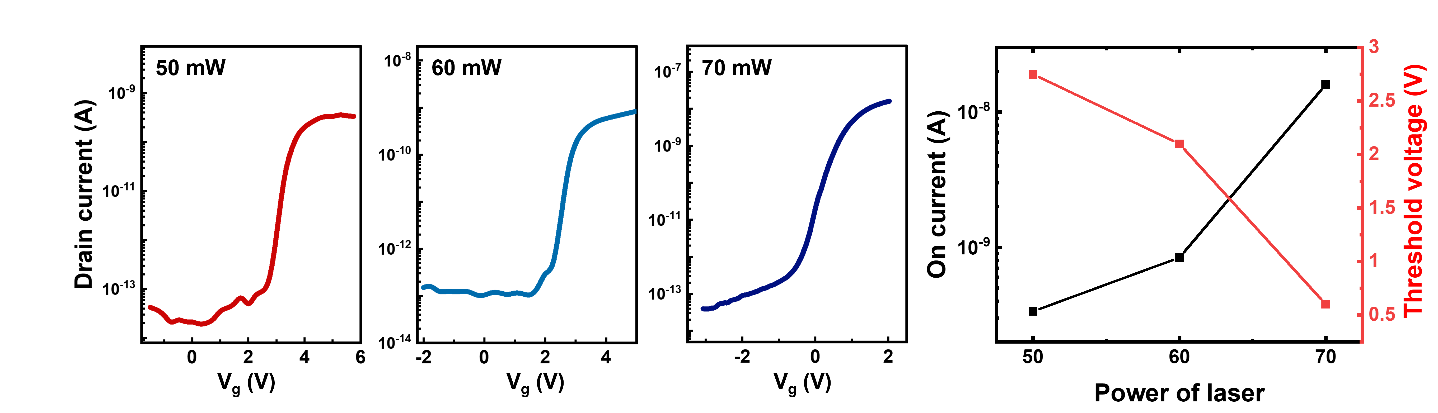
**

**Figure S6.** The I-V characteristics of IGZO-HfO_2_(10nm) following laser annealing at 50, 60 and 70mW power levels with 60μm/s scan speed with compare graph as on current and threshold voltage.

We optimized annealing parameters for IGZO channels using IGZO-HZO stacks. When applying increased laser power densities to an IGZO layer deposited on a 10nm HfO_2_ gate oxide, we noted improved on-current and V_th_ stability, crucial for device reliability. Variations across devices reflect the distinct properties and thermal responses of the HZO and HfO_2_ layers. These differences in material behavior and thermal management during laser annealing significantly impact IGZO channel activation and memory window development.


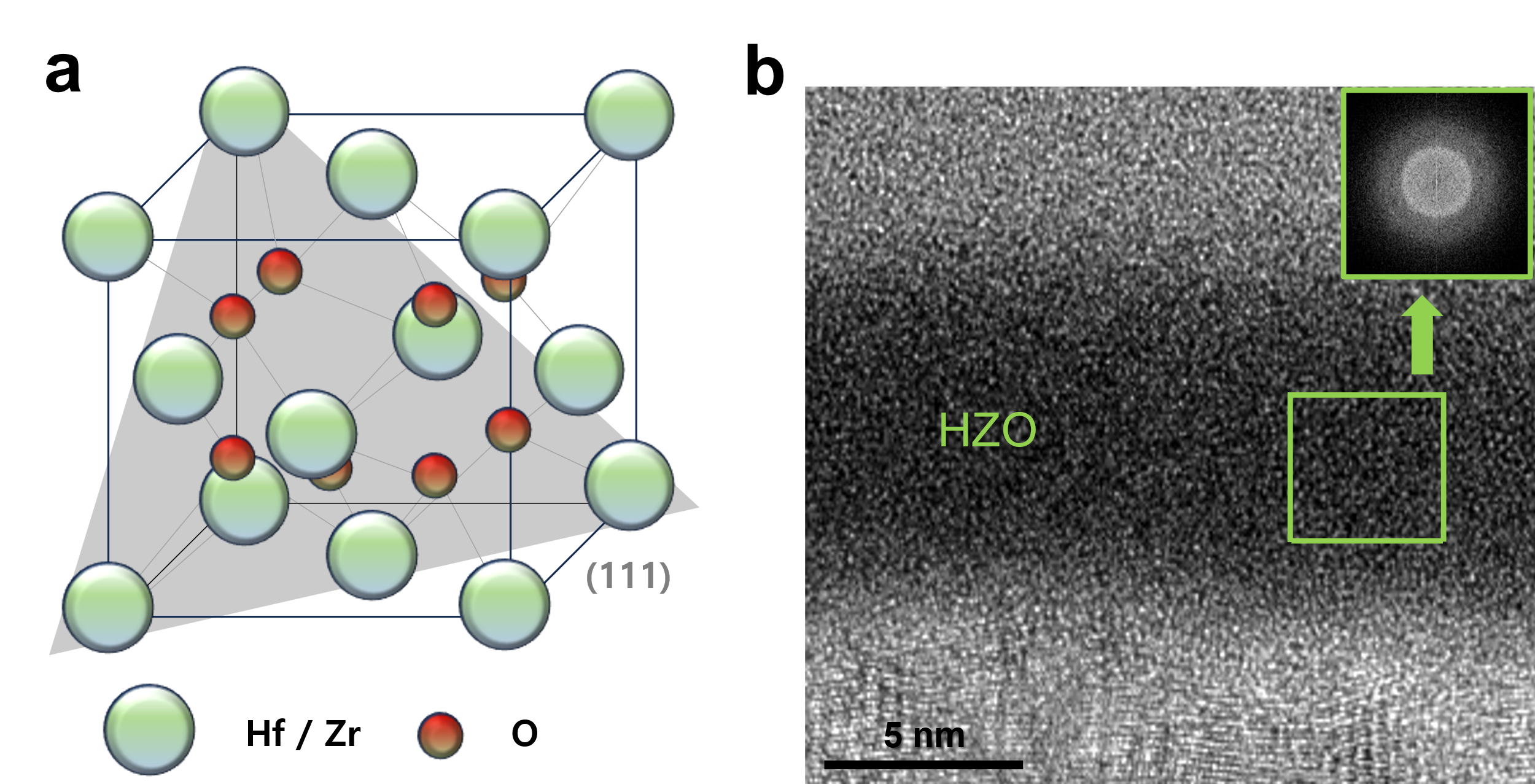
**Structural properties of laser annealing HZO thin films**

**Figure S7.** a) The unit cell of HZO as diffraction planes (111). b) HR-TEM image of an amorphous HZO thin film before laser annealing.

**Figure S7**a a shows the main cells and diffraction planes of the crystal structure of HZO. The orthorhombic phase with ferroelectric properties usually reaches the (111) peak at 30.5° in the XRD data. The case of the analyzed in **Figure 7**e has a lattice distance d=2.92 Å according to the orthorhombic phase of the (111) plane. In the case of **Figure S7**b, which is not annealed, the FFT diffraction picture shows that it is in an amorphous state.

**Enhancing Neuromorphic Functionality in IGZO-HZO FeFETs**

**
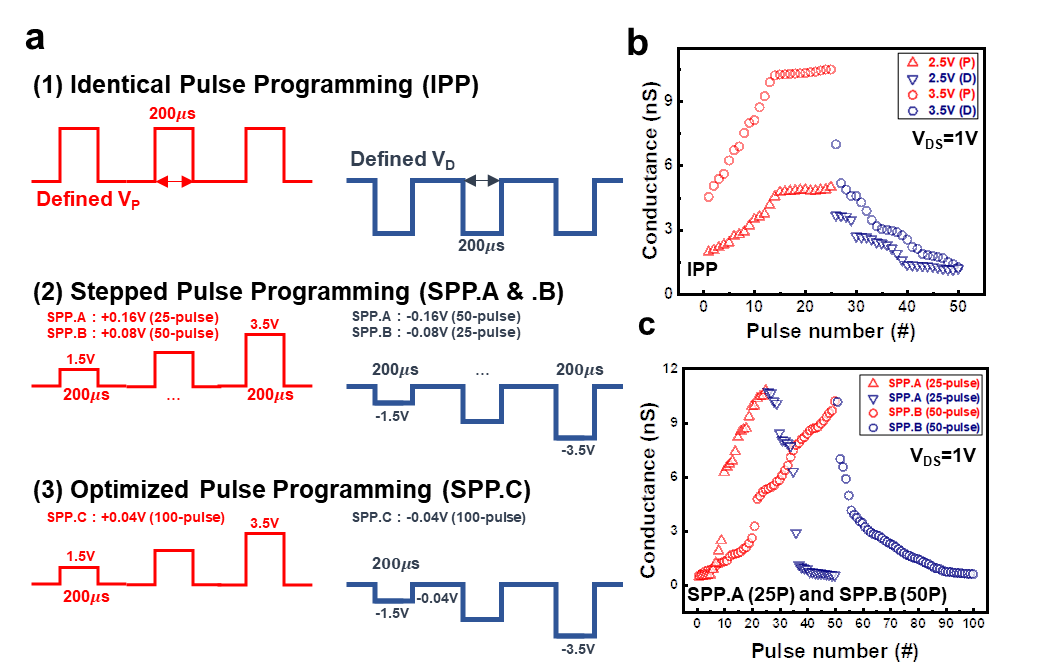
**

**Figure S8.** a) Various gate input pulse schemes for FeFET operation. b) Changes in conductance with the IGZO-HZO FeFET depolarization/polarization with IPP profile. c) Depression and potentiation conductance changes in IGZO-HZO Fe-FET with SPP.A & B pulses at varied increment levels.

This study investigates changes in conductance characteristics to assess the potential of certain devices as memory elements in neuromorphic systems. The conductance of FeFET devices is adjusted using different voltage pulse schemes, based on their coercive and breakdown voltages (1.5 V and 3.7 V, respectively), as shown in **Equation (1)** and **Figure S8**a.

There are three main pulse types:

1. Identical Pulse Programming (IPP) - Uses consistent voltage pulses
2. Stepped Pulse Programming (SPP) - Uses gradually increasing voltage pulses
3. SPP.C - An optimized pulse scheme for this device

The measurement specifically looks at how these pulse types affect conductance range. Consistent pulses offer limited conductance changes, with higher voltages providing broader ranges but making lower-level access difficult, which could impact the reliability of neuromorphic systems. Conversely, incremental pulses schemes allow for complete conductance swings and more precise control over conductance, enhancing system reliability and recognition accuracy without increased power use in **Figure S8**b, c.

**Type 4****. Identical Pulse & Stepped duration Programming (IPP)**

**
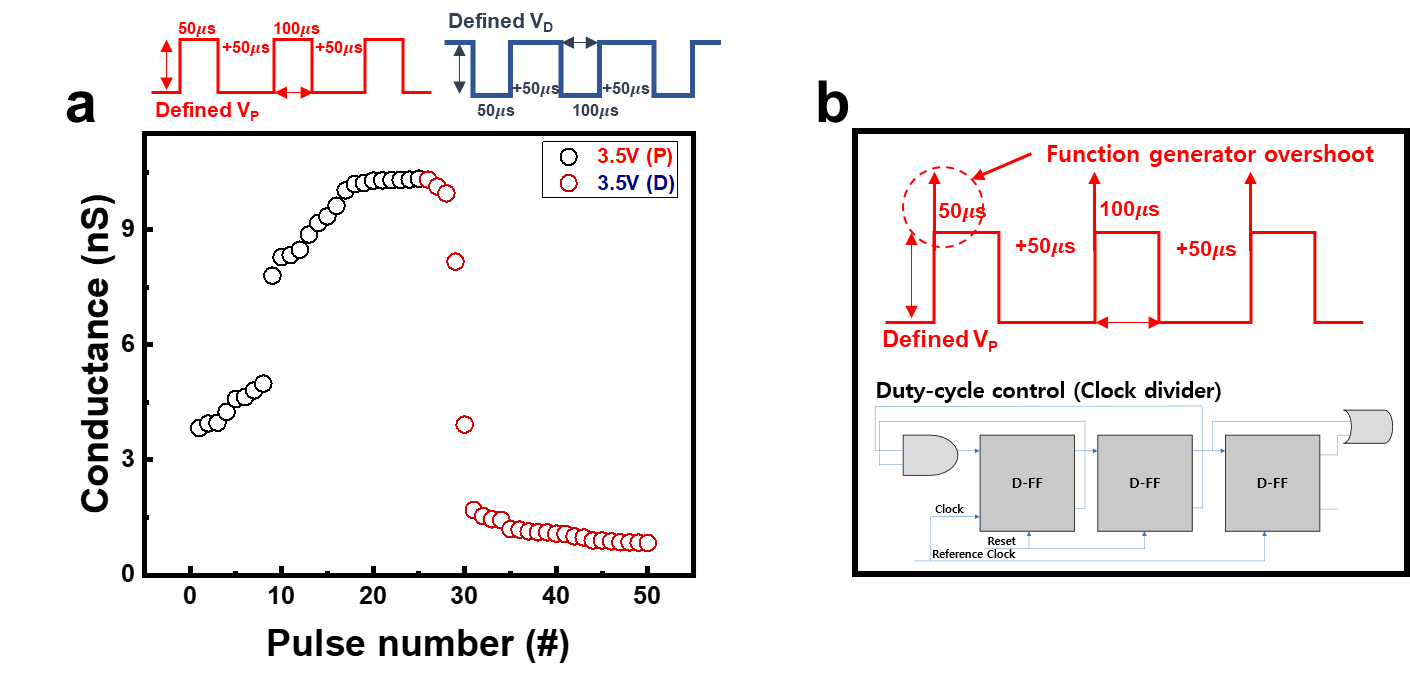
**

**Figure S9**. **Results of conductance change of FeFET device through pulse duration parameter engineering.**

**Figure S9**a shows that HZO-based FeFET was performed by controlling the pulse duration parameter. Depending on the performance of the equipment, a pulse train with an initial value of short duration has linear characteristics in the potentiation of the device. These results show that control through duration also leads to a linear increase in conductance according to the ferroelectric formula. **Figure S9**b shows the overshoot phenomenon of the function generator. Typically, overshoot occurs due to resonance between the inductor and output capacitor. It can reduce this while starting with a very low pulse duty cycle and then increasing it slowly, but this is not a complete solution.[6] Compared to amplitude control, duration control is difficult to control due to complex additional circuits and overshoot precisely.[7] These issues must be considered for the proper operation of neuromorphic devices. In addition, the cost and performance of the circuit system and integration process must be well balanced.

**Linearity calculations for NeuroSIM tools**

In an ideal situation, the increase and decrease of weight values, similar to long-term potentiation (LTP) and long-term depression (LTD), should have a straightforward relationship with the number of pulses used for writing. In NeuroSIM, a model has been created to show how the pulse count (P) affects conductance changes. This model uses the following equation to explain the non-linear behavior of weight updates. [8]

**
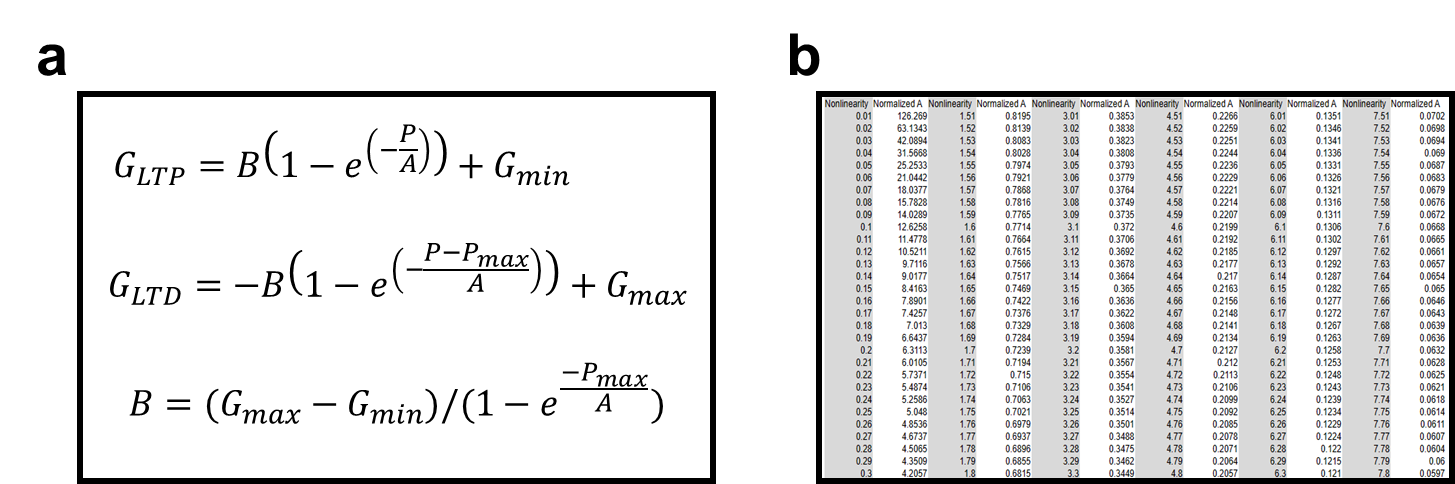
**

**Figure S10**. The formula for calculating linearity a) of neuro sim tool and table b) of linearity fitting parameters.

Here, G_LTP_ and G_LTD_ denote the conductance levels corresponding to Long-Term Potentiation (LTP) and Long-Term Depression (LTD), respectively. The parameters G_max_, G_min_, and P_max_ are derived directly from experimental data, representing the maximum conductance, minimum conductance, and the maximum number of pulses required to transition the device between its minimum and maximum conductance states. In practical simulations, it is noted that these parameters may diverge for LTP and LTD during the fitting process executed by the MATLAB script.[9-10]

**Structural and electrical properties of flexible memory**

**
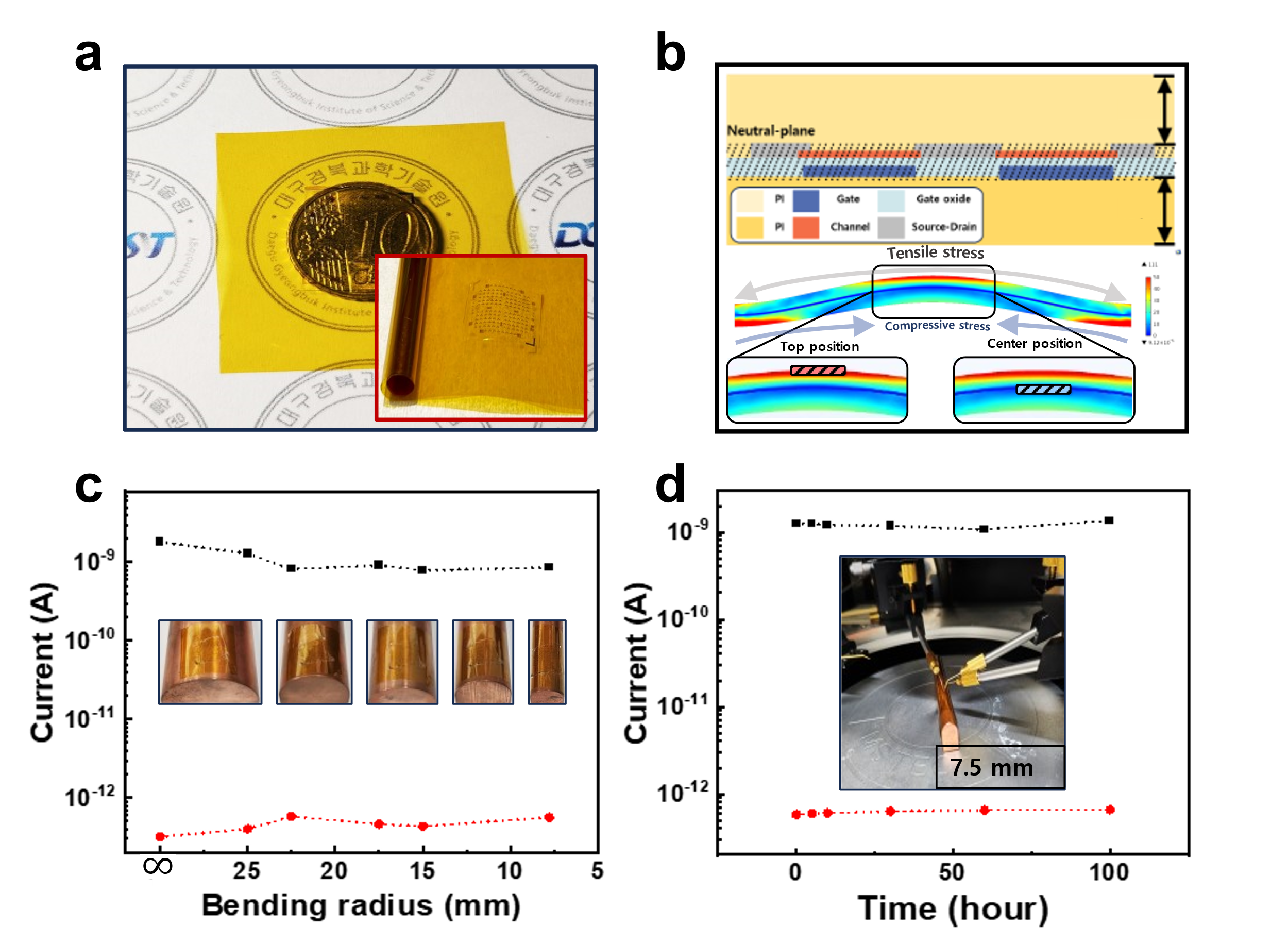
**

**Figure S11**. a) Figure 6a shows an optical picture of a 10-euro cent (R_d_ = 19.75 mm) sized multi-cell IGZO-HZO-FeFET integrated with polyimide to verify the various structure include the rollable form. b) Schematic depicting neutral plane formation under bending stress and strain region analysis results from finite element simulation. c) Variation in on/off behavior with changing bending radius. d) Effect of bending duration on device characteristics with an inset image of the measured device and platform with a bending radius of 7.5 mm.

The PI substrate in this study, shown in **Figure S11**, demonstrated potential as a flexible device. The flexibility of the film is highlighted, with an inset showing the structure in **Figure S11**a. The concept of a neutral plane, where internal stresses cancel each other, is critical for maintaining zero net stress and is discussed in **Figure S11**b. This is important for reducing the PI coating thickness for better integration. The device uses thin film components (IGZO 20 nm, HZO 10 nm) that are well-suited for small neutral layer regions, enhancing device stability in space-constrained applications. Spin-coated PI films allow precise control of thickness under 1 μm. COMSOL simulation confirmed the device's stability within a safe strain zone. To assess flexibility, devices shaped into cylinders with different radii were tested for their on/off functionality at a low gate voltage (V_g_ = 0.1 V), as shown in **Figure S11**c. The results confirmed that the FeFET on/off ratio remained consistent across various bending radii, indicating a stable neutral-plane environment resistant to bending stress. **Figure S11**d illustrates that even under sustained bending, the device maintained consistent performance, highlighting the effectiveness of the neutral layer region as a buffer for device stability.

**Measurement environment settings and programs**

**
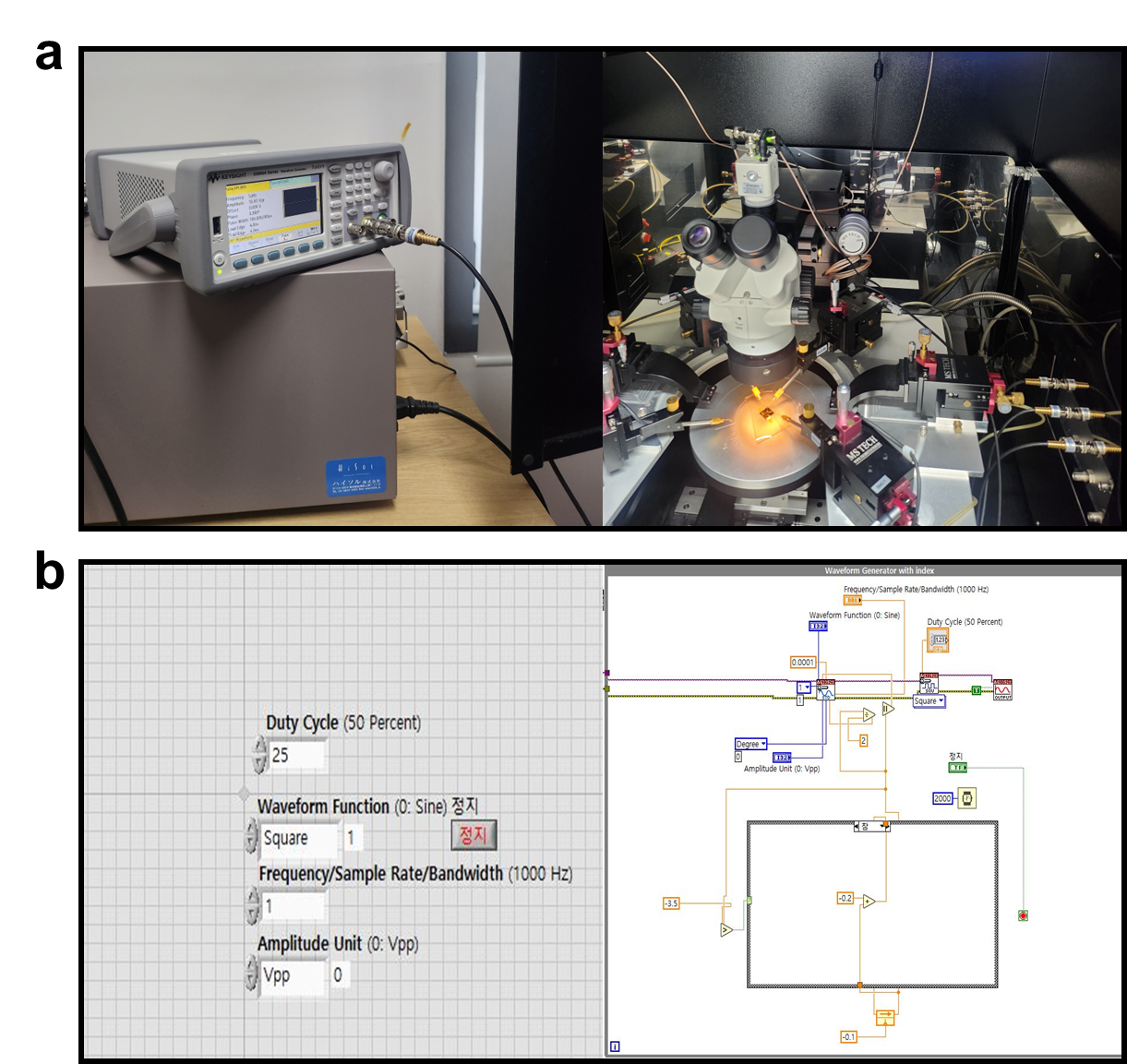
**

**Figure S12**. Establishment of electrical characteristics measurement environment and pulse generation programming a) Measurement setup with Keysight 33250A and Keithley instruments b) LabVIEW pulse programming interface

**Figure S12**a shows the system integrated with the Keysight 33250A and Keithley equipment used for the measurements. Each BNC line is connected to a short box to prevent noise**. Figure S12**b shows the Labview program for creating various pulses. The measurement was optimized by optimizing the delay in the program flow chart.

**Analysis of optical properties of films to extract parameters to be used in simulations**

**
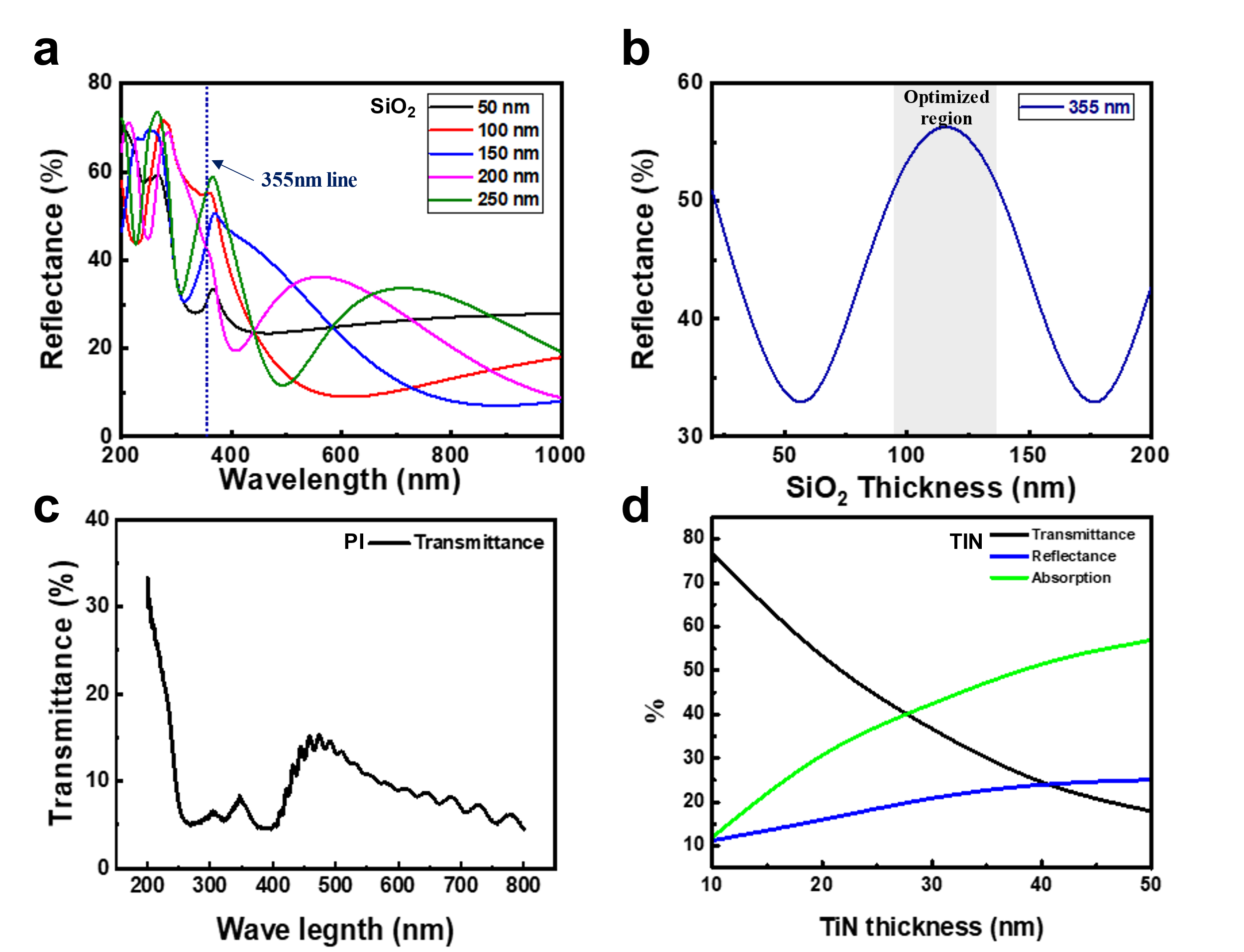
**

**Figure S13**. **Optical property measurement results of each layer for COMSOL simulation.** a) Wavelength-reflectance data by SiO_2_ thickness. b) SiO_2_ thickness-reflectance data at 355 nm wavelength. c) Wavelength-transmittance data of the polyimide used. (d) Transmitance, reflectance, and absorption data by TiN thickness.

The variation in reflectance observed with changes in the thin film's thickness is attributed to the phenomena of multiple refraction and wavelength interference. **Figure S13**a illustrates the reflectance as a function of SiO_2_ thickness, which was determined through fitting with a reflectance calculator based on the Fresnel equation for the 355 nm wavelength employed in this study.[11] As depicted in **Figure S13**b, the reflectance at the 355 nm wavelength exhibits periodic characteristics relative to SiO_2_ thickness. In pursuit of device stability, the optimization region was defined within the vicinity of a thickness of 110 nm. A 100 nm SiO_2_ thickness was identified as the optimal choice, balancing thermal insulation and reflective properties and contributing to enhanced flexibility. The results in **Figure S13**c show that the passage rate of polyimide is very low at 355 nm. This means that the absorption of the laser above PI must be ensured. In **Figure S13**d, it can be seen that the absorbance increases with the thickness of TiN. It can be seen that the absorption of TiN above a certain thickness tends to saturate. Additionally, the laser used has a 30 to 50 nm theoretical depth influence. Accordingly, the thickness of TiN used in this study was optimized to 50 nm.

**Parameters for the materials in the functional layer used in simulation**

**
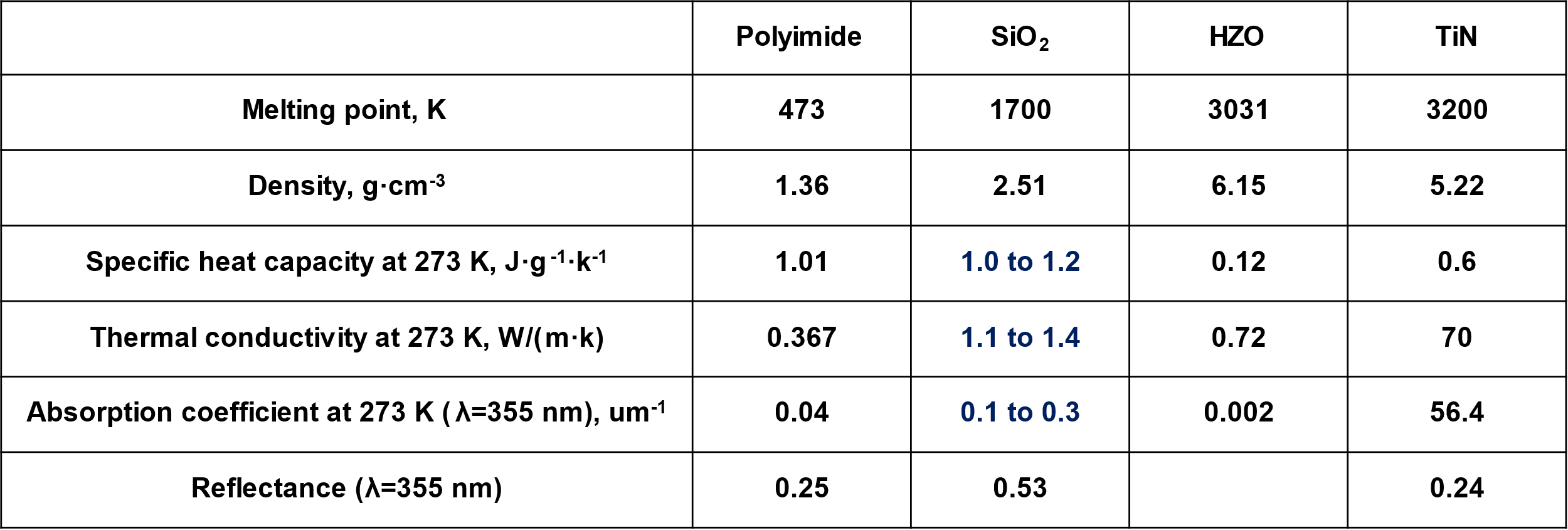
**

**Figure S14.** Parameters for the materials in COMSOL simulation [12-16]

The table shows the parameter measurement and investigation results for thermal and optical Comsol simulations. As temperature increases, it is necessary to consider how the heat capacity, thermal conductivity, and absorption coefficient change by affecting molecules and atoms. It is possible to include these parameters as variables. By predicting the heating and cooling rates of the 355 nm pulse laser through the temperature distribution considering the material parameters, it is possible to select optimal conditions for the crystallization of HZO films and find materials for more sophisticated local annealing processes.

**Reference**

[1] P. Logerais, A. Bouteville, *International Journal of Thermal Sciences* **2010**, 49, 1437.

[2] D. Huang, J. Liu, J. Li, F. Wang, K. Li, Q. Liu, H. Yin, G. Zhang, R. Sun, *ACS Applied Polymer Materials* **2022**, 4, 8508.

[3] H. Lim, H. Kwon, H. Kang, J. E. Jang, H.-J. Kwon, *Nature Communications* **2023**, 14, 3114.

[4] S. Guo, H. Li, Y. Li, Y. Han, K. Chen, G. Xu, Y. Zhu, X. Hu, *Advanced Energy Materials* **2018**, 8, 1800434.

[5] S. Asanuma, K. Sumita, Y. Miyaguchi, K. Horita, T. Masuda, T. Jimbo, N. Miyata, *Applied Physics Express* **2023**.

[6] S. Dong, J. Liu, B. Zhang, C. Lin, C. Zhu, *IEEE Transactions on Power Electronics* **2023**.

[7] J. De Lima, A. Cordeiro, presented at 2001 Conference Proceedings of the 23rd Annual International Conference of the IEEE Engineering in Medicine and Biology Society **2001**.

[8] H.-Y. Chang, P. Narayanan, S. C. Lewis, N. C. Farinha, K. Hosokawa, C. Mackin, H. Tsai, S. Ambrogio, A. Chen, G. W. Burr, *IBM Journal of Research and Development* **2019**, 63, 8: 1.

[9] S. Yu, Y.-C. Luo, T.-H. Kim, O. Phadke, *IEEE Electron Devices Magazine* **2023**, 1, 23.

[10] T.-H. Kim, O. Phadke, Y.-C. Luo, H. Mulaosmanovic, J. Mueller, S. Duenkel, S. Beyer, A. I. Khan, S. Datta, S. Yu, *IEEE Electron Device Letters* **2023**.

[11] H. Zhitao, C. Jinkui, M. Fantao, J. Rencheng, *Journal of Semiconductors* **2009**, 30, 104008.

[12] W. Kim, S. Kim, K.-S. Lee, T. Lee, I. Kim, *Applied surface science* **2012**, 261, 749.

[13] S. Muhammady, Y. Kurniawan, Y. Darma, *Materials Research Express* **2018**, 5, 096303.

[14] R. Krishnan, C. David, P. Ajikumar, R. Nithya, S. Tripura Sundari, S. Dash, B. Panigrahi, M. Kamruddin, A. Tyagi, V. Jayaram, *Journal of Materials* **2013**, 2013.

[15] J. Gong, R. Dai, Z. Wang, C. Zhang, X. Yuan, Z. Zhang, *Materials Research Express* **2017**, 4, 085005.

[16] R. K. Singh, J. Narayan, *Materials Science and Engineering: B* **1989**, 3, 217.
